# Expression Patterns and Identified Protein-Protein Interactions Suggest That Cassava CBL-CIPK Signal Networks Function in Responses to Abiotic Stresses

**DOI:** 10.3389/fpls.2018.00269

**Published:** 2018-03-02

**Authors:** Chunyan Mo, Shumin Wan, Youquan Xia, Ning Ren, Yang Zhou, Xingyu Jiang

**Affiliations:** Hainan Key Laboratory for Sustainable Utilization of Tropical Bioresources, Institute of Tropical Agriculture and Forestry, Hainan University, Haikou, China

**Keywords:** calcineurin B-like protein, CBL-interacting protein kinase, abiotic stress, signal pathway, cassava

## Abstract

Cassava is an energy crop that is tolerant of multiple abiotic stresses. It has been reported that the interaction between Calcineurin B-like (CBL) protein and CBL-interacting protein kinase (CIPK) is implicated in plant development and responses to various stresses. However, little is known about their functions in cassava. Herein, 8 *CBL* (*MeCBL*) and 26 *CIPK* (*MeCIPK*) genes were isolated from cassava by genome searching and cloning of cDNA sequences of *Arabidopsis CBL*s and *CIPK*s. Reverse-transcriptase polymerase chain reaction (RT-PCR) analysis showed that the expression levels of *MeCBL* and *MeCIPK* genes were different in different tissues throughout the life cycle. The expression patterns of 7 *CBL* and 26 *CIPK* genes in response to NaCl, PEG, heat and cold stresses were analyzed by quantitative real-time PCR (qRT-PCR), and it was found that the expression of each was induced by multiple stimuli. Furthermore, we found that many pairs of CBLs and CIPKs could interact with each other via investigating the interactions between 8 CBL and 25 CIPK proteins using a yeast two-hybrid system. Yeast cells co-transformed with cassava *MeCIPK24, MeCBL10*, and Na^+^/H^+^ antiporter *MeSOS1* genes exhibited higher salt tolerance compared to those with one or two genes. These results suggest that the cassava CBL-CIPK signal network might play key roles in response to abiotic stresses.

## Introduction

Calcium is used by most cells to convert external signals into cytosolic information, which can drive processes that are required for full responses to a particular stimulus (Zhai et al., 2013). Therefore, calcium ions play a crucial role as second messengers in mediating various adaptive responses in plants under environmental stresses. Elevation of the cytosolic calcium concentration is a primary event in the responses to many environmental stresses, such as high salinity, drought and cold (Ma et al., 2010). Transient Ca^2+^ change may be sensed by several Ca^2+^-binding proteins including calmodulin (CaM), Ca^2+^-dependent protein kinases (CDPKs) and calcineurin B-like proteins (CBL) (Luan et al., 2002). Such calcium-binding proteins likely function as sensors that recognize changes in calcium parameters and relay these signals into downstream responses, such as phosphorylation cascades and regulation of gene expression (Sanders et al., 2002; Luan et al., 2009).

CBL proteins are important components of three major classes of Ca^2+^ sensors that have been characterized in plants. These proteins are most similar to both the regulatory B subunit of calcineurin (CNB) and neuronal calcium sensors (NCS) in animals. CBL proteins contain an important structural component consisting of four EF-hand domains as calcium-binding sites to capture Ca^2+^ ions (Nagae et al., 2003; Sanchez-Barrena et al., 2005) but do not have enzymatic activity. However, upon Ca^2+^ binding, these proteins interact with their respective target proteins and modulate their activity. The target proteins interacting with CBLs are a family of protein kinases referred to as CBL-interaction protein kinases (CIPKs), which are most similar to the sucrose nonfermenting (SNF) protein kinase from yeast and AMP-dependent kinase (AMPK) from animals in the kinase domain (Batistic and Kudla, 2009; Luan, 2009). Plant SNF1 related kinase (SnRK) have been grouped into three subfamilies: SnRK1, SnRK2, and SnRK3 (Hrabak et al., 1996). SnRK1 plays a role in regulation of carbon and nitrogen metabolism, and SnRK2 and SnRK3 have roles in stress signaling (Shukla and Mattoo, 2008). The CIPK protein, also known as SnRK3, had a conserved NAF/FISL motif in the C-terminal regulatory domain which is required and sufficient for interacting with CBL-type calcium sensors (Albrecht et al., 2001; Guo et al., 2001). Bioinformatics analyses of *Arabidopsis* genome sequences showed a complex signaling network comprised 10 CBLs and 26 CIPKs (Drerup et al., 2013). The first CBL-CIPK pathway was identified during screening for the salt overly sensitive (SOS) phenotype in *Arabidopsis*. In this pathway, a protein kinase complex consisting of AtCBL4 (SOS3) and AtCIPK24 (SOS2) was activated by a salt-stress elicited calcium signal, and then the AtCBL4-AtCIPK24 complex regulated Na^+^/H^+^ exchange activity of SOS1 via phosphorylating a serine residue at its C-terminus in *Arabidopsis* plants under salinity stress (Zhu, 2003; Quintero et al., 2011). AtCBL1 regulated positively the response to salt and drought stresses (Albrecht et al., 2003). In contrast, AtCBL9-AtCIPK3 complex negatively regulated the ABA response during seed germination (Pandey et al., 2008). AtCBL2 interacts with AtCIPK11 and negatively regulates a plasmalemma H^+^-ATPase AHA2 (Fuglsang et al., 2007). AtCBL10 interacts with SOS2 and recruits SOS2 to the plasma membrane to activate a plasma membrane Na^+^/H^+^ antiporter (SOS1) in *Arabidopsis* shoots, which is similar to the function of SOS3 in roots (Quan et al., 2007). Ren et al. reported that AtCBL10 could regulate K^+^ homeostasis by directly interacting with AKT1 in *Arabidopsis* (Ren et al., 2013). AtCBL1/CIPK23 or AtCBL9/CIPK23 complexes could activate the K^+^ channel AKT1 in the plasma membrane and increased *Arabidopsis* ability to uptake K^+^ under low K^+^ conditions (Xu et al., 2006). AtCBL3 interacts with AtCIPK9 to regulate K^+^ homeostasis (Liu et al., 2013). AtCIPK24 regulates vacuolar Na^+^/H^+^ and Ca^2+^/H^+^ exchange activities in *Arabidopsis thaliana* to promote salt tolerance (Cheng et al., 2004; Qiu et al., 2004). AtCBL1-AtCIPK7 kinase complex had an important role in the plant cold tolerance (Huang et al., 2011). The over-expression of AtCBL5 conferred salt and osmotic tolerances to transgenic *Arabidopsis* plants (Cheong et al., 2010). A multivalent interacting network comprised of CBL2/3 and CIPK/26 complexes could protect plants from Mg^2+^ toxicity by sequestrating magnesium (Mg^2+^) into the vacuolar (Tang et al., 2015). AtCIPK8 might regulate nitrate transport activity of AtNRT1.1 at the low-affinity phase (Hu et al., 2009). Therefore, CBL-CIPK calcium signal pathways play vital roles in plant responses to abiotic stresses. Recently, CBL and CIPK families have been identified in other species, including a total of 10 CBLs and 30 CIPKs in rice (Kolukisaoglu et al., 2004), 7 CBLs and 23 CIPKs in canola (Zhang et al., 2014), and 7 CBLs and 29 CIPKs in wheat (Sun et al., 2015). However, except for *Arabidopsis*, the studies on the functions of CBL and CIPK proteins from other plants are still quite limited.

Cassava (*Manihot esculenta*) is one of the most important crop plants. As a food security crop, it provides nourishment for 800 million people around the tropical and sub-tropical world (Oliveira et al., 2014). Cassava is tolerant to environmental stresses such as drought and heat (Zeng et al., 2014). However, the reports about cassava response to abiotic stresses are rare. Therefore, to understand the mechanisms of cassava responses to abiotic stresses, we cloned the *CBL* (*MeCBL*) and *CIPK* (*MeCIPK*) family genes from cassava and analyzed their expression patterns under different abiotic stresses. Furthermore, we systematically studied the interactions between MeCBLs and MeCIPKs. Through this work, we are attempting to establish the CBL-CIPK network in cassava responses to abiotic stress.

## Materials and methods

### Identification of *CBL* and *CIPK* family gene*s* in cassava

The protein sequences of 10 CBLs and 26 CIPKs from *Arabidopsis* (Kolukisaoglu et al., 2004; Drerup et al., 2013) were used as queries to search against the cassava genomic DNA database (http://www.phytozome.net/cassava) using BLASTP with *E*-value less than 1E-5. The putative cassava CBL and CIPK proteins were further compared with the CBLs and CIPKs from *Arabidopsis* and rice by constructing a phylogenetic tree using the Neighbor-Joining (NJ) method. *MeCBLs* and *MeCIPKs* were then named and classified via referring to their orthologous genes from *Arabidopsis* and rice using bootstrap replicates of the Maximum Likelihood phylogenetic tree with values higher than 70 as previously described (Yu et al., 2007).

### Plant growth and gene cloning

Cassava cultivar SC8 (*Manihot esculent* Crantz cv. SC8) plants were grown in the field under natural conditions. The roots, stems, young leaves, old leaves, flowers and storage roots from mature plants were collected and immediately stored at −80°C for the RNA extractions. Meanwhile, the explants were cut from the mother plants, and cultivated on the Murashige and Skoog (MS) medium to induce seedlings in a greenhouse with a 16 h/35°C day and 8 h/20°C night, and a relative humidity of 70%. Forty-day-old seedlings in MS medium were treated with 200 mM NaCl or 20% PEG (polyethylene glycol 6000) at normal temperature. For temperature treatment, the seedlings were cultured under 42° and 4°C conditions. The roots and leaves were harvested at different time intervals (0, 3 and 9 h) and stored at in −80°C immediately for the RNA extraction.

Total RNA was extracted from the *M. esculent* plants using an RNA extraction kit (Tiangen, China). Complementary DNA (cDNA) was synthesized with total RNA as template using the PrimeScript RT reagent kit (TaKaRa, Japan). The gene specific primers of *MeCBL* and *MeCIPK* genes were designed using Primer Premier 5 software. Primers used in this study are shown in Table S1. *MeCBLs* and *MeCIPKs* were amplified by polymerase chain reaction (PCR) from cDNA mixtures. The PCR amplification conditions were initiated at 94°C for 5 min, followed by 35 cycles of 94°C for 30 s, 50°–60°C (depends on the TM value of gene-specific primers) for 30 s, 72°C for 1 min per kilo-base pair (kb), then a final extension at 72°C for 10 min. The PCR products were then examined by electrophoresis and sequenced.

### Bioinformation analysis

The isoelectric point (pI) and molecular weight (MW) of each MeCBL and MeCIPK protein were predicted using the ExPASy tool (http://web.expasy.org/protparam/). The palmitoylation sites and myristoylation sites were predicted by CSS-Palm 3.0 (http://csspalm.biocuckoo.org/). Predictions of motifs were generated using MEME (Multiple Em for Motif Elicitation) program (http://meme-suite.org/tools/meme). Gene structures of MeCBLs and MeCIPKs were analyzed using the Gene Structure Display Server (GSDS 2.0, http://gsds.cbi.pku.edu.cn/). Sequence alignments were predicted by the DNAMAN software. The classification and naming of MeCBL and MeCIPK proteins were performed as described in the above section “Identification of *CBL* and *CIPK* Family Gene*s* in Cassava.” The *cis*-acting elements in the 2,000 bp upstream sequences of coding region of cassava *MeCBL* and *MeCIPK* genes (http://www.phytozome.net/cassava) were analyzed using the PlantCARE software (http://bioinformatics.psb.ugent.be/webtools/plantcare/html/) as previously reported (Xi et al., 2017).

### Gene expression analysis

To analyze the tissue specificity of *MeCBL* and *MeCIPK*, the expression levels in different tissues were examined by semi-quantitative RT-PCR. The housekeeping gene *Actin* was used as an internal control. The primers are shown in Table S1. The PCR conditions were as follows: 95°C for 5 min; 95°C for 45 s, 56°C for 30 s, 72°C for 45 s for 28 cycles; and a final extension of 72°C for 5 min. The PCR products were examined on 2% agarose gel and photographed under UV light.

Forty-day-old seedlings were treated with different stresses as described above. Then, the roots and leaves were collected. Quantitative real-time PCR (qRT-PCR) was performed using an ABI 7900HT system (TaKaRa, Japan). The qRT-PCR amplification conditions were as follows: 95°C for 1 min, followed by 45 cycles at 95°C for 5 s and 60°C for 30 s. A dissociation curve from 60° to 95°C was generated to verify the primer specificity. The relative expression levels were calculated by the 2^−ΔΔCT^ method. Three replicate biological experiments were conducted. Primers used in this study are shown in Table S1.

### Yeast two-hybrid assays

The MatchMaker yeast two-hybrid system (Clontech, USA) was used to examine protein interactions. Firstly, the *MeCBL* genes were inserted into the pGBKT7 vector and the *MeCIPK* genes were cloned into the pGADT7 vector. Primers used in this study are shown in Table S1. Then, the plasmids were transformed into the yeast strain Y2HGold according to the Yeast Protocols Handbook (Clontech). After screening on SD medium lacking leucine and tryptophan (SD-L-T, DDO), the positive clones were examined using PCR. Subsequently, the positive clones were incubated in DDO medium at 28°C for 1 day. Aliquots (10 μL) were spotted onto non-selective medium (DDO) and selective medium (lacking leucine, tryptophan, histidine and adenine, SD-L-T-H-A, QDO) supplemented with 40 μg/mL X-α-Gal and 125 ng/mL aureobasidin A and incubated for 5 days before being photographed.

### Yeast complementation test

The coding sequence of cassava Na^+^/H^+^ antiporter gene *MeSOS1* was obtained from the cassava genomic DNA database (http://www.phytozome.net/cassava) using *AtSOS1* sequence as a query (Quan et al., 2007; Quintero et al., 2011), And then the *MeSOS1* gene was inserted into the yeast expression vector pYPGE15. The full length coding regions of genes *MeCIPK24* and *MeCBL10* were cloned by PCR using the primers shown in Table S1 and then inserted into the yeast expression vector p414, respectively. The three plasmids (p414-*MeCIPK24*, p414-*MeCBL10* and pYPGE15-*MeSOS1*) were co-transformed into the yeast strain AXT3K (*ena1*:: *HIS3*:: *ena4, nha1*::*LEU2*, and *nhx1*::*KanMX4*) lacking the endogenous NHX1 protein, the plasma membrane Na^+^ efflux transporters NHA1 and the sodium pumps ENA1-4 according to the previous report (Zhou et al., 2015). The positive clones were screened on YNB medium (0.17% yeast nitrogen base without amino acids, 0.5% ammonium sulfate, 2% glucose) and yeast complementation tests were analyzed on AP medium (0.174% arginine, 2% glucose, 8 mM H_3_PO_4_, 2 mM MgSO_4_, 0.2 mM CaCl_2_, 1 mM KCl, 1 × trace elements and 1 × vitamins) with different NaCl concentrations (Zhou et al., 2015). After incubation for 5 days at 28°C, the growth was imaged and analyzed.

### Statistics analysis

The real-time PCR data were determined with the SDS plate utility software version 2.4. Data were analyzed using Microsoft Excel and Statistical Package for the Social Sciences (Chicago, IL, USA). The means were separated using Student's *t*-test at the 5% level of significance.

## Results

### Identification of *CBL* and *CIPK* family genes in cassava

In order to identify the *CBL* and *CIPK* genes from cassava, 10 CBL and 26 CIPK protein sequences from *Arabidopsis* were used as queries to run BLAST searches using the cassava genomic DNA database (http://www.phytozome.net/cassava). As a result, 8 CBLs (MeCBL1 to MeCBL10, except for MeCBL3 and MeCBL7) and 26 CIPKs were identified and named based on the similarities to *Arabidopsis* orthologs with *Me* standing for *Manihot esculent* (Table 1). The detailed information, including protein length, isoelectric point (pI), molecular weight (MW), palmitoylation sites and myristoylation sites, of the identified MeCBLs and MeCIPKs is listed in Table 1. The MW of the predicted MeCBL proteins ranged from 24.50 to 28.74 kD and of the MeCIPK proteins ranged from 40.94 to 56.05 kD.

**Table 1:** Features of *CBL* and *CIPK* genes in cassava.

**Gene name**	**Locus name**	**Arabidopsis ortholog/AGI No**.	**Protein length**	**pI**	**MW (kD)**	**Palmitoylation sites Amino acid (location)**	**Myristoylation sites Amino acid (location)**
MeCBL1	cassava4.1_016071m.g	AtCBL1/At4g17615	213	4.62	24.56	C (3)	G (2)
MeCBL2	cassava4.1_023888m.g	AtCBL2/At5g55990	223	4.77	25.84	C (4)	G (7)
MeCBL4	cassava4.1_015878m.g	AtCBL4/At5g24270	218	5.20	25.36	C (8), C (10)	–
MeCBL5	cassava4.1_022392m.g	AtCBL5/At4g01420	225	4.60	25.94	C (8), C (10)	G (7)
MeCBL6	cassava4.1_014733m.g	AtCBL6/At4g16350	248	4.70	28.56	–	–
MeCBL8	cassava4.1_023193m.g	AtCBL8/At1g64480	214	4.78	24.51	C (7)	G (6)
MeCBL9	cassava4.1_016083m.g	AtCBL9/At5g47100	213	4.76	24.50	C (3)	G (2)
MeCBL10	cassava4.1_014701m.g	AtCBL10/At4g33000	249	4.70	28.74	C (20)	–
MeCIPK1	cassava4.1_008050m.g	AtCIPK1/At3g17510	431	7.15	48.47	–	G (4), G (9)
MeCIPK2	cassava4.1_007534m.g	AtCIPK2/At5g07070	449	8.37	51.35	–	G (5)
MeCIPK3	cassava4.1_010345m.g	AtCIPK3/At2g26980	362	5.85	40.94	–	–
MeCIPK4	cassava4.1_008191m.g	AtCIPK4/At4g14580	427	9.12	47.53	C (218)	–
MeCIPK5	cassava4.1_029811m.g	AtCIPK5/At5g10930	448	8.69	50.74	–	–
MeCIPK6	cassava4.1_031025m.g	AtCIPK6/At4g30960	438	9.19	49.43	C (236)	G (2), G (8)
MeCIPK7	cassava4.1_008412m.g	AtCIPK7/At3g23000	420	9.07	47.08	–	–
MeCIPK8	cassava4.1_007600m.g	AtCIPK8/At4g24400	446	7.63	50.53	–	G (7)
MeCIPK9	cassava4.1_007907m.g	AtCIPK9/At1g01140	437	8.94	49.29	–	–
MeCIPK10	cassava4.1_007266m.g	AtCIPK10/At5g58380	459	8.30	51.94	C (231)	–
MeCIPK11	cassava4.1_034294m.g	AtCIPK11/At2g30360	433	9.10	49.13	C (179)	–
MeCIPK12	cassava4.1_006117m.g	AtCIPK12/At4g18700	499	6.63	56.05	–	–
MeCIPK13	cassava4.1_024110m.g	AtCIPK13/At2g34180	450	9.12	50.94	–	G (5)
MeCIPK14	cassava4.1_008116m.g	AtCIPK14/At5g01820	429	7.08	48.24	–	–
MeCIPK15	cassava4.1_006767m.g	AtCIPK15/At5g01810	476	8.92	53.95	–	G (5)
MeCIPK16	cassava4.1_028375m.g	AtCIPK16/At2g25090	453	8.66	50.87	–	G (5)
MeCIPK17	cassava4.1_007161m.g	AtCIPK17/At1g48260	463	8.79	51.89	–	G (5), G (10)
MeCIPK18	cassava4.1_007849m.g	AtCIPK18/At1g29230	438	9.05	49.11	C (236)	G (8)
MeCIPK19	cassava4.1_006740m.g	AtCIPK19/At5g45810	477	8.08	53.63	–	–
MeCIPK20	cassava4.1_025576m.g	AtCIPK20/At5g45820	454	9.20	51.32	–	G (7)
MeCIPK21	cassava4.1_006970m.g	AtCIPK21/At5g57630	469	6.08	52.97	C (379), C (383)	G (2)
MeCIPK22	cassava4.1_030445m.g	AtCIPK22/At2g38490	398	9.02	44.72	–	–
MeCIPK23	cassava4.1_007136m.g	AtCIPK23/At1g30270	463	8.95	51.77	C (302)	G (5)
MeCIPK24	cassava4.1_007604m.g	AtCIPK24/At5g35410	447	8.29	50.17	C (441)	–
MeCIPK25	cassava4.1_007324m.g	AtCIPK25/At5g25110	457	8.79	51.76	–	–
MeCIPK26	cassava4.1_007827m.g	AtCIPK26/At5g21326	439	6.88	50.26	–	–

*The nomenclature of MeCBL and MeCIPK followed that of Arabidopsis CBL and CIPK proteins through a comparison using MEGA 5.0 software. The protein length, isoelectric point (pI) and molecular weight (MW) of the encoded proteins were predicted by ExPASy tool (http://web.expasy.org/protparam/). The palmitoylation sites and myristoylation sites were predicted by CSS-Palm 3.0 (http://csspalm.biocuckoo.org/), G represent the glycine residue, and C represent the cysteine residue*.

**Figure 1:**
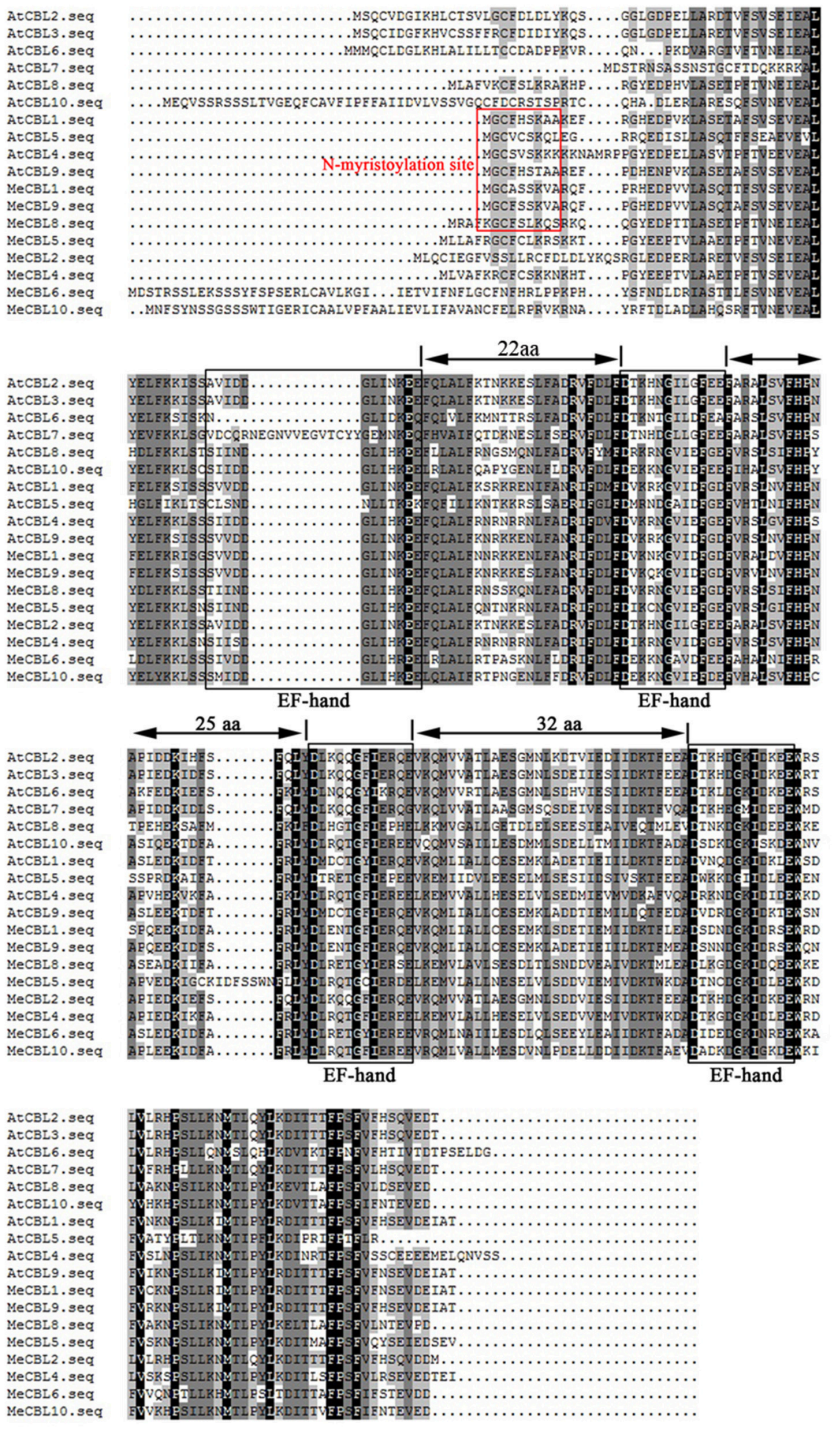
Multiple sequence alignment between cassava and *Arabidopsis* CBL proteins. Sequence alignment was performed using DNAMAN 5.0 software. Identical amino acids are shaded in black, and similar amino acids are shaded in gray. The four EF hand motifs are indicated by black boxes. The myristoylation sites are in the red box.

Furthermore, sequence alignments of the multiple amino acids between MeCBLs and AtCBLs are shown in Figure 1. The results indicate that the sequences of MeCBLs are highly conserved: all the MeCBLs containing four EF hand motifs, which are similar to the AtCBLs EF-hand motifs. The MeCBL proteins also have conserved linkers between each EF motif. There are 22 amino acids between EF1 and EF2 domains, 25 amino acids between EF2 and EF3 domains, and 32 amino acids between EF3 and EF4 domains (Figure 1). However, there are 32 amino acids between EF2 and EF3 domains in the MeCBL5 protein (Figure 1). In addition, seven CBLs have palmitoylation sites, but MeCBL6 does not; five CBLs have a myristoylation site in the N-terminal domain, but MeCBL4, MeCBL6 and MeCBL10 do not (Figure 1, Table 1).

**Figure 2:**
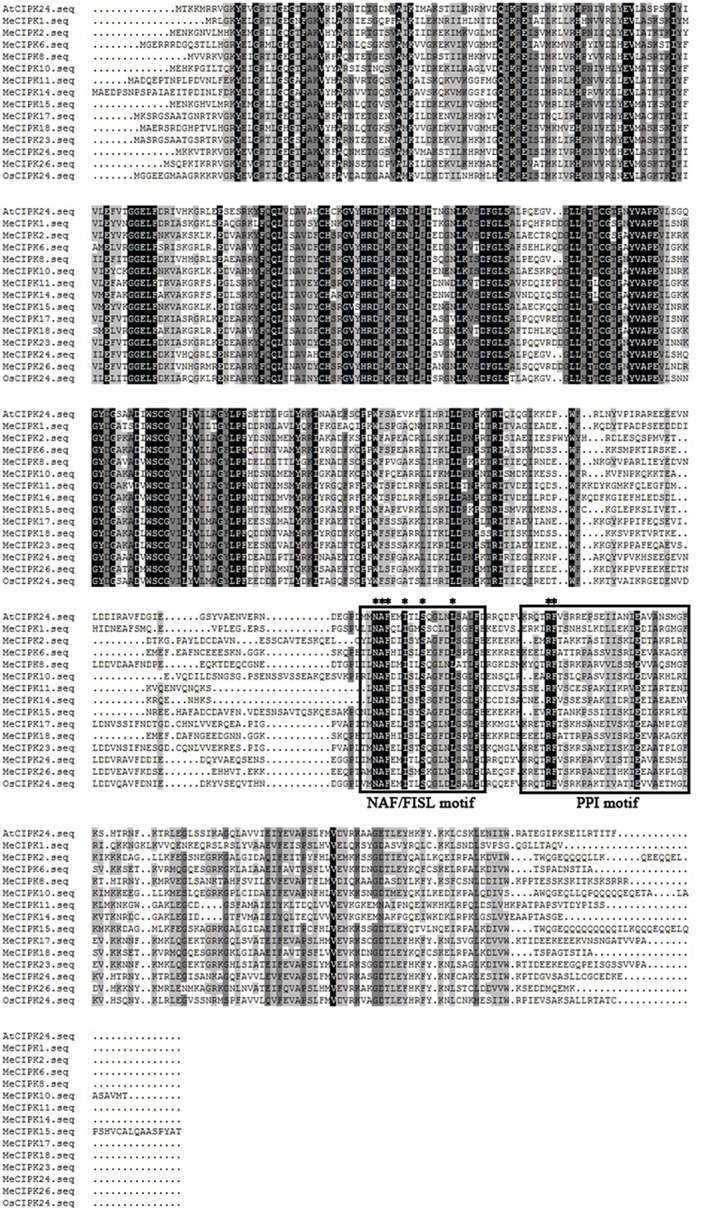
Multiple sequence alignment between cassava and *Arabidopsis* CIPK proteins. Sequence alignment was performed using DNAMAN 5.0 software. Identical amino acids are shaded in black, and similar amino acids are shaded in gray. The NAF/FISL and PPI motifs are indicated by black boxes. The conserved amino acids in the motifs are indicated by asterisk.

Similarly, the alignment results showed that all the MeCIPKs contain an N-terminal catalytic kinase domain and a C-terminal regulatory domain, which are jointed by a variable domain. The NAF/FISL motif is conserved in all the MeCIPKs (Figure 2). This motif has been reported to be necessary for mediating interactions between CIPK and CBL proteins (Albrecht et al., 2001; Guo et al., 2001). Sequence analysis also showed that a protein-phosphatase interaction (PPI) motif is conserved in the C-terminus of the kinases (Figure 2). In addition, eight CIPKs, including MeCIPK4, 6, 10, 11, 18, 21, 23, and 24, have palmitoylation sites. Nine CIPKs, including MeCIPK2, 13, 15, 16, 17, 18, 20, 21, and 23, have a myristoylation site in the N-terminal domain. MeCIPK1, 6 and 8 have two myristoylation sites in the N-terminal domain (Table 1).

### Phylogenetic analysis of cassava CBL and CIPK proteins

To investigate the evolutionary history between cassava CBL and CIPK proteins and other species, a Neighbor-Joining phylogenetic tree was constructed using CBL and CIPK protein sequences from cassava, *Arabidopsis* and rice. The CBL and CIPK family proteins were clustered into four (Figure S1) and five (Figure S2) groups, respectively. The results showed that MeCBL6 and MeCBL10 are clustered in group I and were identified as orthologous with AtCBL10 and OsCBL10. MeCBL2 is homologous to AtCBL2 in group II. In group III, MeCBL1 and MeCBL9 are close to AtCBL1 and AtCBL9. MeCBL4 and MeCBL5 are similar in sequence and homologous to AtCBL4, and MeCBL8 is close to AtCBL8. MeCBL4 and MeCBL5 formed in Group IV. A phylogenetic tree showed that the CIPK family contains five groups (Figure S2). Group A includes MeCIPK1, 3, 8, 9, 17, 21, 22, 23, 24, and 26. Group B contains MeCIPK4, 6, 7 and 18. Group C contains MeCIPK2, 5, 10, 13, 15, 16, 20, and 25. Group D contains MeCIPK11 and MeCIPK14. Group E contains MeCIPK12 and MeCIPK19.

In addition, closely-related orthologous pairs of CBLs and CIPKs were identified between cassava and *Arabidopsis*, with bootstrap values higher than 80, such as for MeCBL8 and AtCBL8 (Figure S1), MeCIPK8 and AtCIPK8, MeCIPK21 and AtCIPK21, MeCIPK24, and AtCIPK24 (Figure S2). These results suggest that an ancestral set of *CBL* and *CIPK* genes existed prior to the divergence of cassava and *Arabidopsis*.

### Gene structure and conserved motifs of cassava MeCBLs and MeCIPKs

**Figure 3:**
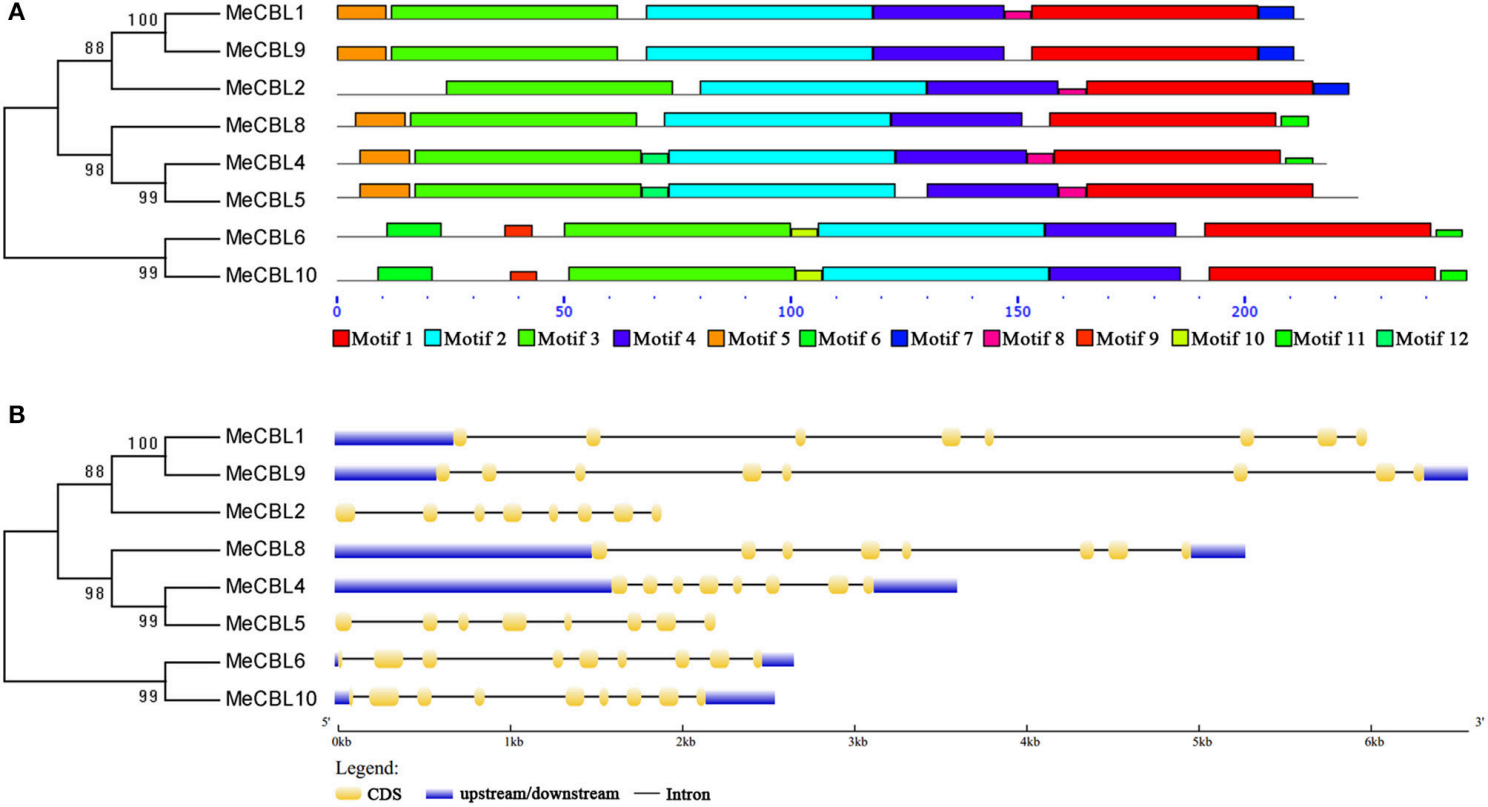
Conserved motifs **(A)** and gene structures **(B)** of cassava CBL proteins and genes, respectively. **(A)** The conserved motifs were identified using the MEME program and are arranged according to the phylogenetic tree. Different motifs are highlighted with different color boxes. The length of boxes corresponds to motif length. **(B)** The gene structures were drawn using the GSDS program and arranged according to the phylogenetic tree. The yellow boxes represent exons, the blue boxes represent upstream and downstream UTRs and the lines represent introns.

In order to investigate the structural features of cassava CBL and CIPK genes and proteins, intron/exon organization and conserved motifs were investigated by GSDS and MEME software, respectively. As shown in Figure 3A and Figure S3A, there are twelve motifs in MeCBL proteins. All MeCBL proteins contain motif 1 to motif 4, which were annotated as the four EF-hand domains (Figure 1, Figure S3A). Motif 10 is only found in group I, including MeCBL6 and MeCBL10, and the motif 11 is only present in MeCBL4 and MeCBL5 belonging to Group IV, which suggests that these motifs play specific roles in the corresponding groups. Furthermore, the intron/exon structural analyses revealed that all the *MeCBL* genes contain seven introns, except that genes in group I have eight introns (Figure 3B).

**Figure 4:**
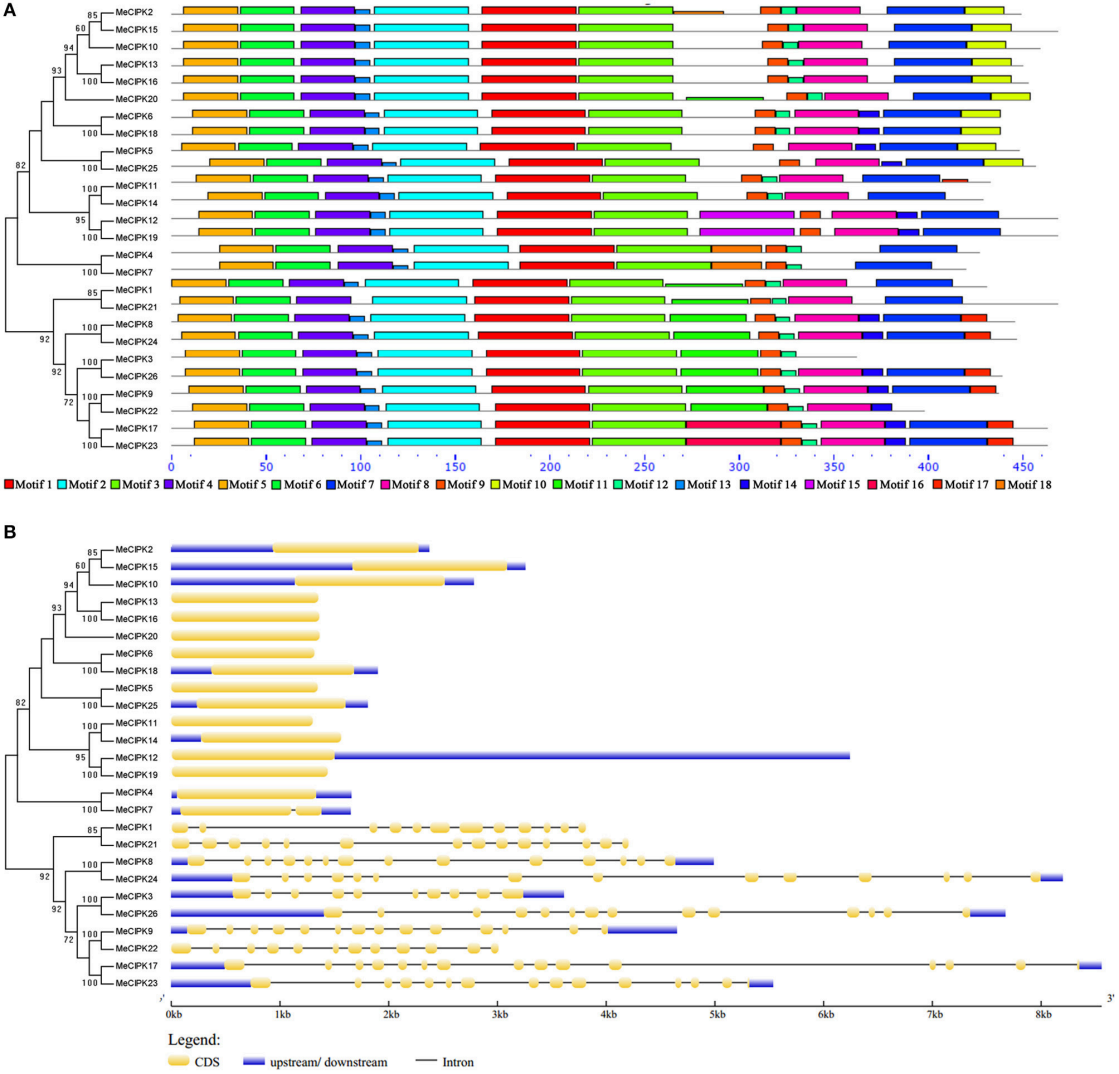
Conserved motifs **(A)** and gene structures **(B)** of cassava CIPK genes. **(A)** The conserved motifs were using the MEME program and arranged corresponding to the phylogenetic tree. Different motifs are highlighted with different color boxes. The length of boxes corresponded to motif length. **(B)** The gene structures were drawn using the GSDS program and arranged corresponding to the phylogenetic tree. The yellow boxes represent exons, the blue boxes represent upstream and downstream UTRs and the lines represent introns.

Eighteen motifs were identified in MeCIPK proteins (Figure 4A, Figure S3B). Of them, motif 9 is the NAF/FISL domain and it is widely distributed in all MeCIPKs. Motif 8, which is annotated as a PPI domain for phosphatase interaction, is also widely distributed in MeCIPK proteins except for MeCIPK3, MeCIPK4 and MeCIPK7 (Figure 4A). Furthermore, the GSDS software predicted that the intron-rich *MeCIPK* genes cluster in group A, but the number of introns varied from nine to thirteen. Genes in the other four groups (B, C, D and E) have no introns except that *MeCIPK7* has one intron (Figure 4B).

### Promoter analysis of *MeCBL* and *MeCIPK* genes

To analyze the possible regulatory mechanisms of transcription of *MeCBL* and *MeCIPK* genes, the *cis*-acting elements in 2,000 bp of the immediate upstream sequences of the coding regions of *MeCBL* and *MeCIPK* genes were searched using the PlantCARE software. Besides the common CAAT-box and TATA-box elements, sixty-nine potential *cis*-acting elements were detected and divided into four types according to their biological functions (Table S2). The first type is light response-related elements such as the CT1-motif, the GT1-motif, the ATC-motif, the GATT-motif and the CG-motif. The Box 4 element was detected in all the 8 *CBL* genes with the most being detected in the *MeCBL6* gene (6 of the Box 4 type), *MeCBL10* gene (5 of the Box 4 type) and *MeCBL9* gene (5 of the Box 4 type). Similarly, the Box 4 element was detected in most *CIPK* genes except for *MeCIPK8* and *MeCIPK26*, which had the most numbers in *MeCIPK12* gene (16 of the Box 4 type) and *MeCIPK20* gene (11 of the Box 4 type). The second type is hormone-responsive elements such as the ABRE element involved in the abscisic acid response, the CGTCA-motif involved in the MeJA-response, the ethylene-responsive element ERE and the gibberellin-responsive element GARE-motif. The *cis*-acting element CE1 type involved in ABA responsiveness was only detected in *MeCIPK6* and *MeCIPK18*. The third type is plant development-related elements such as the GCN4-motif involved in endosperm expression, the RY-element involved in seed-specific regulation, the CCGTCC-box related to meristem specific activation and HD-Zip 1, which is involved in differentiation of the palisade mesophyll cells. The Skn-1 motif, *cis*-acting regulatory element required for endosperm expression, was detected in all the 8 *CBL* genes with the most being detected in the *MeCBL2* gene (4 of the Skn-1 motif) and *MeCBL5* gene (4 of the Skn-1 motif). The MBSI element involved in flavonoid biosynthetic genes regulation was only detected in *MeCBL9* gene. The last type is abiotic stress related elements. The HSE element involved in the heat stress response was detected in *MeCBL6, 8, 9*, and *10*, and all *MeCIPKs* except *MeCIPK1, 2, 3, 4, 11* and *24*. The LTR element involved in low-temperature response was detected in *MeCBL5, 8* and *9* and *MeCIPK1, 3, 9, 10*. The MBS element containing the MYB binding site involved in the drought response was detected in *MeCBL1, 4, 5*, and *8* and all *MeCIPKs* except *MeCIPK5, 6, 7, 10, 12, 17, 20, 21, 24*, and *25*. The TC-rich repeats involved in defense and some stress responses was detected in all the *MeCBLs* and *MeCIPKs* except *MeCIPK2, 8, 12, 13*, and *17* (Table S2).

### Expression analyses of *MeCBL* and *MeCIPK* family genes

**Figure 5:**
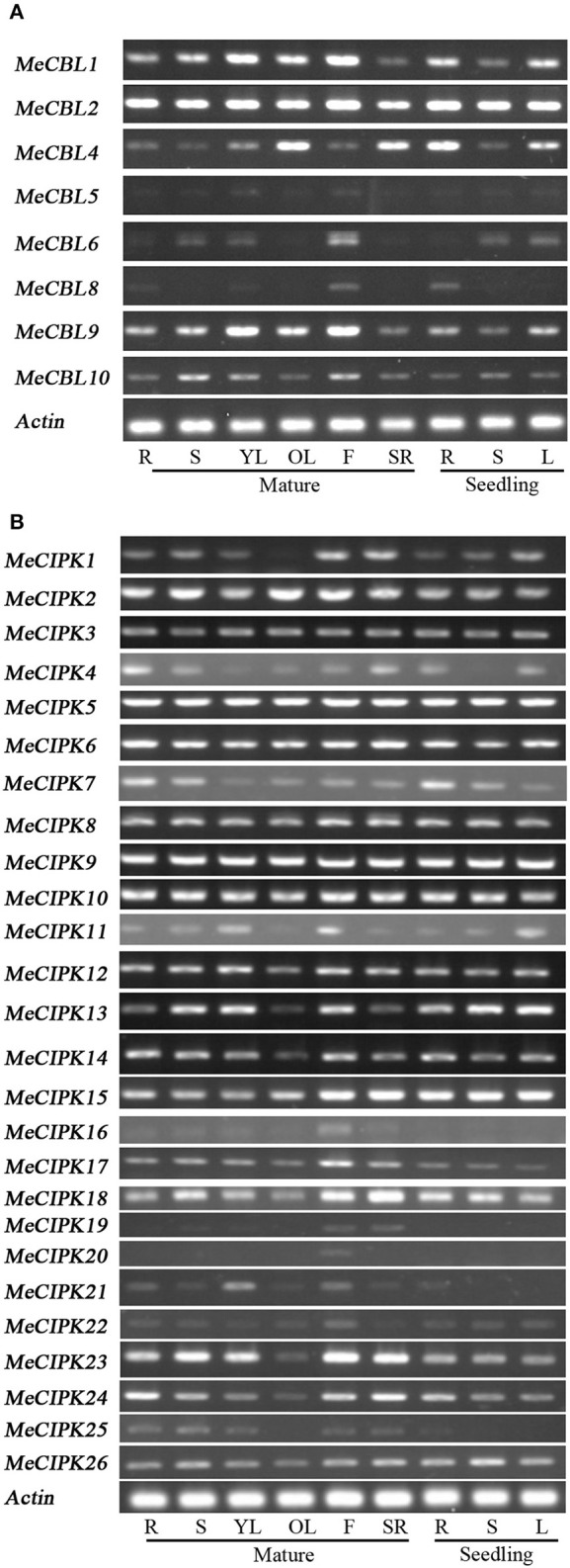
Expression profile analyses of *MeCBL* **(A)** and *MeCIPK* **(B)** genes in cassava tissues using the RT-PCR method. “R” represents roots from mature plants or seedlings, “S” represents stems from mature plants or seedlings, “YL” and “OL” represent young leaves and old leaves of mature plants, respectively, “F” represents flowers, “SR” represents storage roots, and “L” represents the seedlings leaves.

To investigate the spatial expression patterns of *MeCBLs* and *MeCIPKs* in cassava, transcript levels were studied using RT-PCR in different tissues of seedlings and mature plants, including root (seedling and mature stage), stem (seedling and mature stage), leaf (seedling stage, and mature stage: young leaves and old leaves), flower and storage root (Figure 5). The results show that the expression levels of most of *MeCBLs* and *MeCIPKs* are different in all tissues tested. However, some genes such as *MeCBL2, MeCIPK5, 6, 9*, and *10* were constitutively expressed in all tissues and at all developmental stages. Some genes were mainly expressed in specific organs. For example, *MeCIPK16* and *MeCIPK20* were mainly expressed in flower, indicating that these genes might have specific roles in this organ. Other genes, like *MeCBL5, MeCIPK16, 19*, and *20* had very low transcript levels in the tested organs. Moreover, the gene expression levels in seedlings are different from mature plants, for example, *MeCBL1, MeCBL9*, and *MeCIPK23* have higher transcriptional levels in the mature stage than that in the seedling stage (stems and leaves).

**Figure 6:**
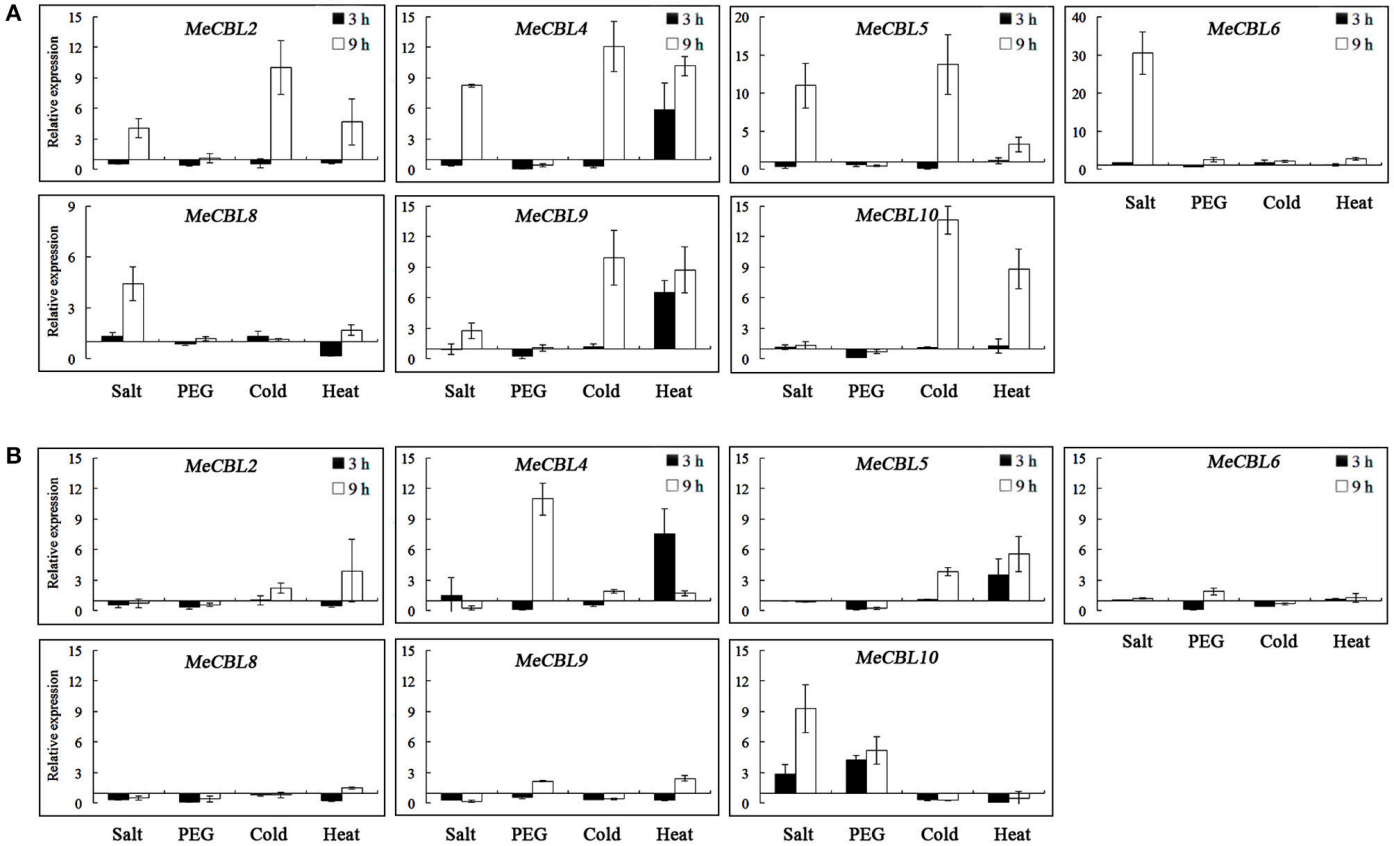
Expression analyses of *MeCBL* genes in roots **(A)** and leaves **(B)** responding to abiotic stresses, including salt (200 mM NaCl), drought (20% PEG6000), cold (4°C) and heat (42°C) treatments. Data are the means of three biological replicates ± SE at 3 h and 9 h time points standardized at 0 h.

**Figure 7:**
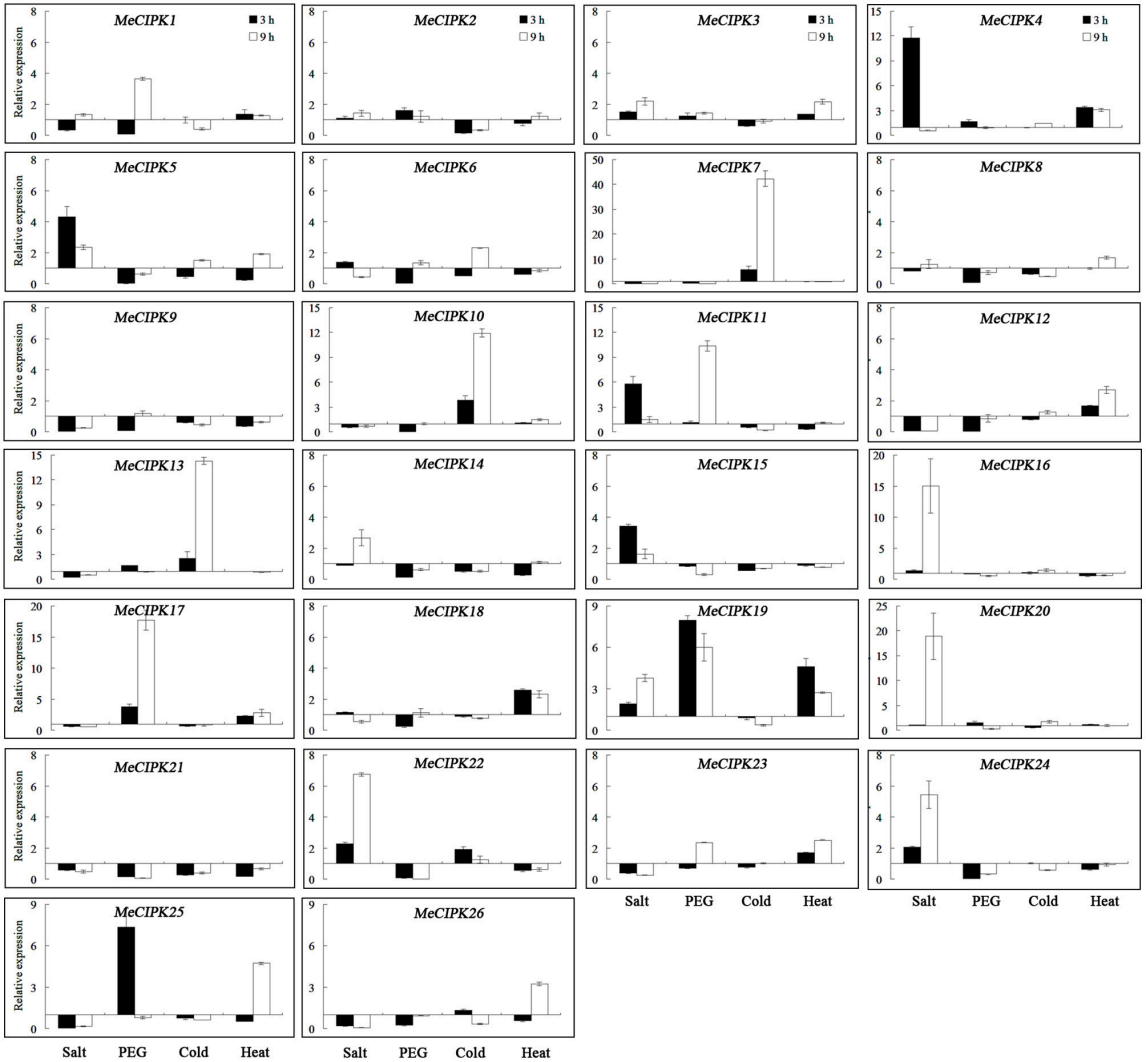
Expression analyses of *MeCIPK* genes in roots responding to abiotic stresses, including salt (200 mM NaCl), drought (20% PEG6000), cold (4°C) and heat (42°C) treatments. Data are the means of three biological replicates ± SE at 3 and 9 h time-point standardized at 0 h.

**Figure 8:**
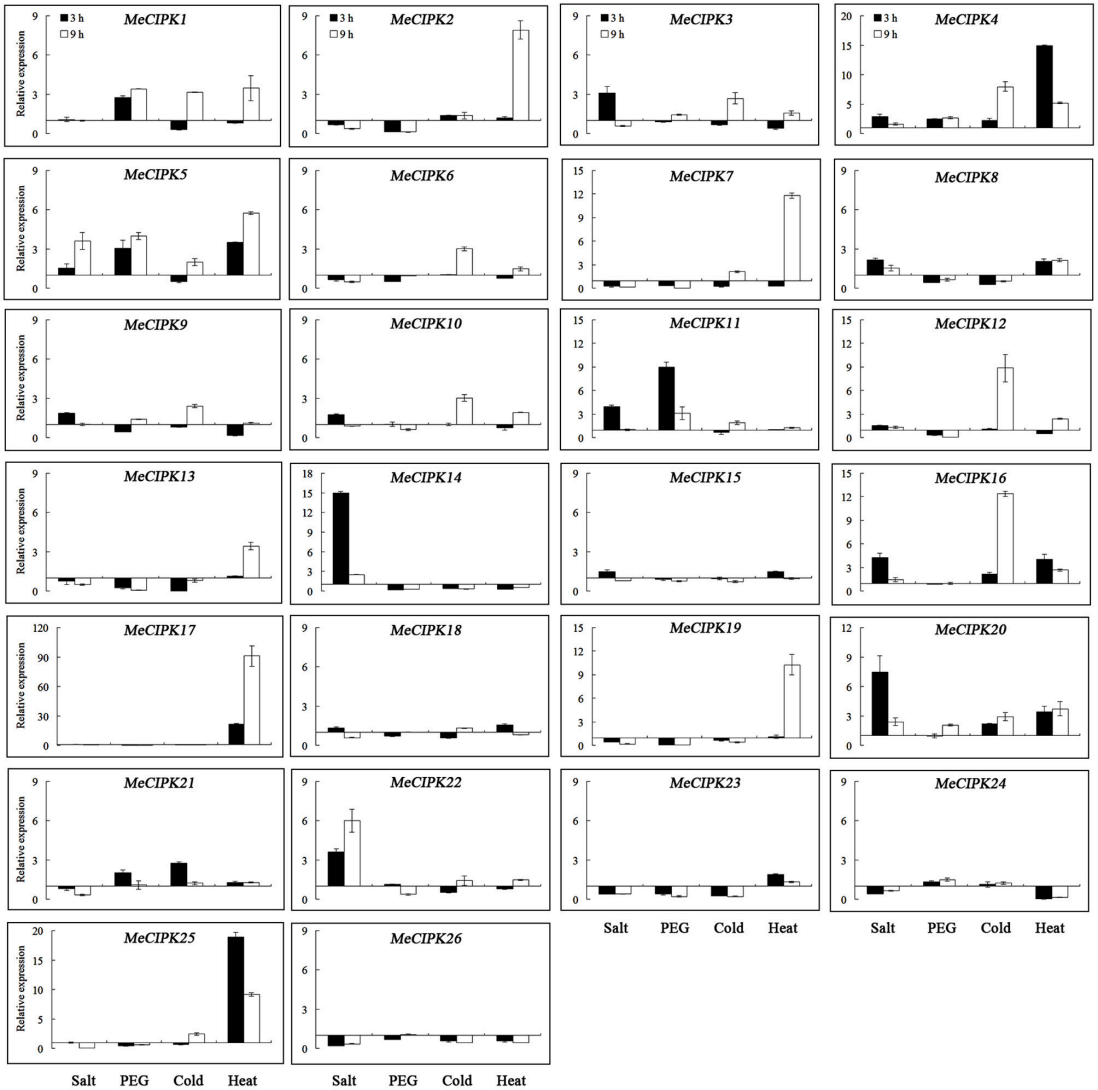
Expression analyses of *MeCIPK* genes in leaves responding to abiotic stresses, including salt (200 mM NaCl), drought (20% PEG6000), cold (4°C) and heat (42°C) treatments. Data are the means of three biological replicates ± SE at 3 h and 9 h time-point standardized at 0 h.

It has been reported that the *CBL* and *CIPK* genes play significant roles in response to abiotic stresses (Chen et al., 2011; Yu et al., 2014; Zhang et al., 2014; Sun et al., 2015; Xi et al., 2017). Therefore, forty-day-old cassava seedlings were subjected to stresses including salt (200 mM NaCl), drought (20% PEG6000), cold (4°C) and heat (42°C) treatment and the expression profiles of *MeCBL* and *MeCIPK* genes were investigated using qRT-PCR. The levels of all the MeCBLs and MeCIPKs were altered under stress treatments, and the transcript levels of 7 *MeCBLs* and 26 *MeCIPKs* in roots and leaves are shown in Figures 6–**8**. Under 200 mM NaCl treatment, *MeCBL6* and *MeCBL8* in roots were up-regulated at both 3 h and 9 h time-points. *MeCBL4* and *MeCBL5* transcript levels showed up-regulation at the 9 h time-point, but these genes were down-regulated at 3 h after salt treatment of roots (Figure 6A). *MeCBL10* was the only up-regulated gene in leaves upon salt stress (Figure 6B). In PEG-treated cassava seedlings, *MeCBL4* and *MeCBL10* were up-regulated in leaves at 9 h after treatment, but the other genes did not significantly change at any time point tested in roots (Figure 6A) or leaves (Figure 6B). In addition, *MeCBL2, 4, 5, 9*, and *10* were all induced by high temperature or cold stress in roots (Figure 6A). *MeCBL4* and *MeCBL5* in leaves were up-regulated at both 3 and 9 h time-points when treated with high temperature stress (Figure 6B).

As for the *MeCIPK* genes assayed, *MeCIPK4, 5, 11, 16, 20, 22*, and *24* in roots and *MeCIPK14* and *20* in leaves were up-regulated at the 3 h time-point by salt stress (Figures 7, 8). In 9 h PEG-treated cassava roots, *MeCIPK11, 17, 19*, and *25* were up-regulated and expression of the *MeCIPK7, 14, 21, 22*, and *24* were down-regulated (Figure 7). However, only *MeCIPK11* was induced in leaves after PEG treatment (Figure 8). *MeCIPK7* was significantly induced in roots by cold treatment. *MeCIPK10* and *13* in roots (Figure 7) and *MeCIPK12* and *16* in leaves (Figure 8) were also affected after cold treatment. The expression of *MeCIPK19* in roots (Figure 7) and *MeCIPK2, 4, 17*, and *25* in leaves (Figure 8) was induced by heat treatment at both time-points. Some genes like *MeCIPK8, 9, 15, 18*, and *26* were not affected significantly by NaCl, PEG, cold and heat stresses, but might be induced by other abiotic stresses. It is worth noting that *MeCIPK24*, the ortholog of *AtCIPK24* (*AtSOS2*) which regulates salt tolerance via activating Na^+^/H^+^ exchange activity of AtSOS1 in *Arabidopsis* (Quintero et al., 2011), was only up-regulated in roots exposed to salt stress. So the gene was chosen for further salt tolerant assays.

The expression profiles of *MeCBLs* and *MeCIPKs* in cassava exposed to various stress conditions suggests that different CBLs and CIPKs may participate in the same signaling process and play roles in abiotic stresses.

### Interaction analyses of MeCBL and MeCIPK proteins

Many reports have demonstrated that some CIPK proteins interact with specific CBL proteins in response to environment stresses (Xu et al., 2006; Ho et al., 2009; Tang et al., 2015; Wang et al., 2016). To investigate the interaction preferences of MeCBL and MeCIPK proteins, the yeast two-hybrid system was used. Eight *MeCBL*s and 25 *MeCIPK*s were cloned and inserted into the pGBKT7 and pGADT7 vectors, respectively, and then transformed into the yeast strain Y2HGold. The interaction relationships between MeCBL and MeCIPK proteins were detected by yeast growth on non-selective medium (DDO) and selective medium (QDO+X+). As shown in Table 2 and Figure S4, MeCBL4 could interact with eight CIPKs (MeCIPK2, 7, 10, 14, 16, 18, 19, and 22), and MeCBL5 could interact with seven CIPKs (MeCIPK3, 4, 8, 10, 14, 17, and 19), the two CBL proteins have been identified as orthologs of AtCBL4 (Figure S1). MeCBL10, orthologous with AtCBL10 and OsCBL10 (Figure S1), showed strong interaction with eight CIPKs (MeCIPK1, 5, 8, 18, 19, 22, 23, and 24). In contrast, MeCIPK6 and MeCIPK11 could not interact with any of the eight MeCBL proteins in this study, suggesting that they might participate in other signaling pathways with other unidentified MeCBL proteins in cassava. MeCIPK12 only interacted with MeCBL2, MeCIPK20 only interacted with MeCBL6 and MeCIPK25 only interacted with MeCBL9, which suggests that some CIPK proteins interact only with a specific CBL protein. As for MeCIPK24, an ortholog of *Arabidopsis* AtCIPK24 (AtSOS2), it could strongly interact with MeCBL2, 6, and 10, which suggests that MeCIPK24-MeCBL2, MeCIPK24-MeCBL6, and MeCIPK24-MeCBL10 might take part in regulating salt tolerance in cassava.

**Table 2:** Interaction of MeCBLs and MeCIPKs in yeast two-hybrid assay.

	**MeCBL1**	**MeCBL2**	**MeCBL4**	**MeCBL5**	**MeCBL6**	**MeCBL8**	**MeCBL9**	**MeCBL10**
MeCIPK1	−	−	−	−	+	+	+	+
MeCIPK2	+	+	+	−	+	−	−	−
MeCIPK3	−	−	−	+	+	−	−	−
MeCIPK4	−	−	−	+	+	−	−	−
MeCIPK5	−	−	−	−	+	−	−	+
MeCIPK6	−	−	−	−	−	−	−	−
MeCIPK7	−	+	+	−	+	−	−	−
MeCIPK8	+	−	−	+	+	−	+	+
MeCIPK9	−	−	−	−	+	−	+	−
MeCIPK10	+	+	+	+	+	+	+	−
MeCIPK11	−	−	−	−	−	−	−	−
MeCIPK12	−	+	−	−	−	−	−	−
MeCIPK13	−	+	−	−	+	−	−	−
MeCIPK14	+	−	+	+	+	+	+	−
MeCIPK15	+	+	−	−	+	+	−	−
MeCIPK16	+	+	+	−	+	+	+	−
MeCIPK17	+	−	−	+	−	−	−	−
MeCIPK18	+	−	+	−	+	+	+	+
MeCIPK19	+	−	+	+	+	+	+	+
MeCIPK20	−	−	−	−	+	−	−	−
MeCIPK22	−	−	+	−	+	−	−	+
MeCIPK23	+	+	−	−	+	−	+	+
MeCIPK24	−	+	−	−	+	−	−	+
MeCIPK25	−	−	−	−	−	−	+	−
MeCIPK26	+	−	−	−	+	+	+	−

*The interaction analyses of MeCBL and MeCIPK proteins were performed using yeast two-hybrid system. Eight MeCBLs and 25 MeCIPKs was cloned and inserted into the pGADT7 and pGBKT7 plasmid, respectively. The plasmids were transformed into the yeast strain Y2HGold through lithium acetate method, and the interacting relationships were detected by the yeast growth on non-selective medium (DDO: SD-T-L) and selective medium (QDO+X+AbA: SD-T-L-H-A+40 μg/mL X-α-Gal + 125 ng/mL Aureobasidin A). + represents growth (interaction), − represents no growth (no interaction)*.

### Co-expression *CBL10* and *CIPK24* improves salt tolerance in transgenic yeast

**Figure 9:**
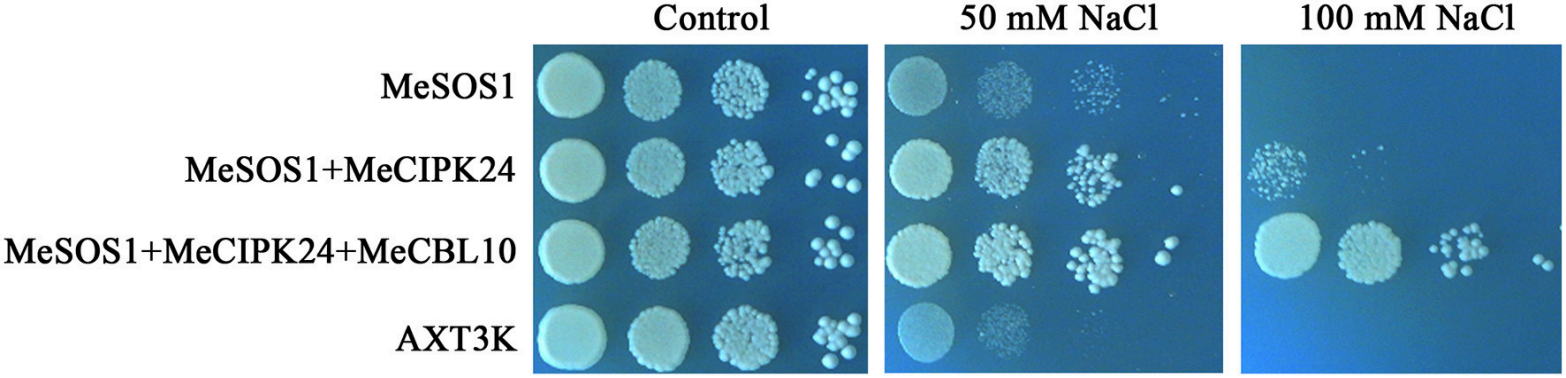
The effect of salinity stress on yeast growth. Transgenic and untransformed yeast cells were pre-cultured to saturation, and serial 10-fold dilutions of yeast cells were spotted on AP plates without or with NaCl as described. After 5 days, the growth of yeast cells on plates was recorded. MeSOS1, AXT3K strain transformed with the Na^+^/H^+^ antiporter gene *MeSOS1*; MeSOS1+MeCIPK24, AXT3K strain transformed with *MeSOS1* and *MeCIPK24* genes; MeSOS1+MeCIPK24+MeCBL10, AXT3K strain transformed with *MeSOS1, MeCIPK24* and *MeCBL10* genes; AXT3K, untransformed AXT3K strain.

CBL10 is a calcium sensor and CIPK24/SOS2 is a protein kinase that, together with SOS1, are the three key components comprising the salt tolerance signaling pathway identified in *Arabidopsis*. The CBL10-CIPK24 complex activates the Na^+^/H^+^ exchange activity of SOS1 to extrude Na^+^ out of cells during salt stress (Quan et al., 2007). The SOS signaling pathway has been demonstrated to be conserved in *Arabidopsis*, rice and poplar plants, and SOS-like proteins from these three distantly related plants could form inter-species protein complexes and regulate salt tolerance of transgenic yeast cells (Martinez-Atienza et al., 2007; Tang et al., 2010). As seen from Figure 9, *MeCBL10* and *MeCIPK24* are orthologs of *AtCBL10* and *AtCIPK24* in *Arabidopsis* and were up-regulated by salt stress in cassava (Figures 6, 7). Also, the yeast two-hybrid assay showed that MeCBL10 could interact with MeCIPK24 (Table 2, Figure S4), so theoretically the MeCBL10-MeCIPK24 complex could be involved in cassava salt tolerance by regulating the Na^+^/H^+^ antiport activity of SOS1. To test the hypothesis, *MeCBL10, MeCIPK24*, and *MeSOS1* were co-transformed into a yeast mutant strain AXT3K. Functional analyses indicated that the co-expression of three genes conferred stronger salt tolerance to transgenic yeast cells than *MeCIPK24*-*MeSOS1* co-transgenic or single *MeSOS1* transgenic cells. These results suggest that the SOS pathway, comprised of MeCBL10, MeCIPK24 and MeSOS1, is conserved, and that a MeCBL10-MeCIPK24 signal pathway regulates cassava salt tolerance together with the plasma membrane Na^+^/H^+^ antiporter SOS1.

## Discussion

Calcium participates in the signal transduction response to various environmental stimuli. As a calcium sensor, CBL protein, often work with it target kinase, CIPK protein, to regulate plant response to abiotic stresses (Kudla et al., 2010). CBL-CIPK signaling networks have been studied in many plants, such as *Arabidopsis*, canola, grapevine, poplar, rice, wheat, and other plants (Kolukisaoglu et al., 2004; Xiang et al., 2007; Zhang et al., 2008, 2014; Sun et al., 2015; Xi et al., 2017). But there are few studies in cassava at present.

Multiple alignments showed that all the CBL proteins contained four EF hand motifs, which are necessary for CBL proteins to bind Ca^2+^ (Nagae et al., 2003; Sanchez-Barrena et al., 2005). The EF-hand domains are less conserved and may contribute functional diversity, while the linkers between each EF motif are absolutely conserved in CBL proteins (Zhang et al., 2008). In this study, 8 *CBL* and 26 *CIPK* genes were identified from the cassava genome. The EF-hand motifs are organized in fixed spaces that are often 22, 25, and 32 amino acids between EF1 and EF2, EF2 and EF3, EF and EF4 domains, respectively, except for MeCBL5, in which there were 32 amino acids between EF2 and EF3 domains (Figure 1). However, phylogenetic analysis of MeCBL5 did indicate that it has high homology with *Arabidopsis* AtCBL4 protein (Figure S1).Therefore, MeCBL5 might have a specific function, but this needs to be investigated further. Furthermore, five MeCBLs, including MeCBL1, 2, 5, 8, and 9, were determined to have a myristoylation site in the N-terminus, which is a Ca^2+^-binding domain (Du et al., 2011). The CIPK proteins have been demonstrated to contain two domains: the N-terminal kinase catalytic domain and the C-terminal regulatory domain harboring the NAF/FISL motif and PPI motif. The NAF/FISL motif is necessary for interaction between CIPK and CBL proteins (Guo et al., 2001). In the present study, all the MeCIPKs possess a NAF domain in the C-terminal region except for MeCIPK4, which seems to lack this domain. Because of homology to AtCIPK4 and OsCIPK4 (Figure S2), MeCIPK4 is still considered to be a valid MeCIPK. The PPI motif in AtCIPK24 (AtSOS2) is necessary for interaction with ABI2 (abscisic acid-insensitive 2), a protein phosphatase 2C (Ohta et al., 2003). In the PPI motif in AtCIPK24, the Arg-340 and Phe-341 are important for the kinases to interact with protein phosphatases. When the amino acids were substituted with alanine, the interaction was abolished (Ohta et al., 2003). Sequence alignments showed that arginine and phenylalanine are highly conserved in MeCIPK proteins (Figure 2).

Intron/exon organizations often reflect the evolution of some gene families (Wang et al., 2013; Liu et al., 2014). Most *MeCBL* genes have seven introns, while *MeCBL6* and *MeCBL10* have eight introns (Figure 3B). The phylogenetic tree analysis showed that MeCBL6 and MeCBL10 belong to group I (Figure S1). These suggest that the functions of CBL proteins might be different. As shown in Figure S2, *MeCIPK* genes were divided into five groups. Most interestingly, the members from groups B, C, D and E have fewer introns (and some have no introns) compared to genes from group A, which each contain at least nine introns (Figure 4B). This feature of *CIPK* gene structures was also found in *Arabidopsis*, rice, maize and soybean (Kolukisaoglu et al., 2004; Chen et al., 2011; Zhu et al., 2016), which suggests that intron gain or loss have played important roles in CIPK evolution.

Many researches have demonstrated that CBL and CIPK function in response to environment stress. Loss of *AtCBL1* rendered plants drought sensitive and over-expression of *AtCBL1* reduced transpirational water loss (Albrecht et al., 2003). Over-expression of *AtCBL5* increased salt or osmotic tolerance of transgenic plants (Cheong et al., 2010). *MeCBL5* was up-regulated by salt stress and cold stress in roots. Drought related element MYB and low-temperature response element LTR were also found in the promoter region of *MeCBL5* (Figure 6, Table S2). Transgenic rice over-expressing barley *HsCBL8* showed enhanced salt tolerance (Guo et al., 2016). The orthologous gene *MeCBL8* was up-regulated by salt stress and stress response element TC-rich repeats was found in the *MeCBL8* promoter region (Figure 6, Table S2). *AtCIPK3* was responsive to ABA and cold stress conditions, and, therefore AtCIPK3 might participate in abscisic acid and cold signal transduction in *Arabidopsis* (Kim et al., 2003). The orthologous gene *MeCIPK3* was induced by salt stress and cold in leaves and low-temperature responses element LTR was found in the *MeCIPK3* promoter region (Figure 8, Table S2). AtCIPK8 participates in regulating the low-affinity phase of the primary nitrate response (Hu et al., 2009). However, MeCIPK8 was not significantly affected by the four treatments applied in this study (Figure 7, 8). Additionally, heat treatment induced the expression of *MeCIPK19* in roots (Figure 7) and *MeCIPK7, 17* and *25* in leaves (Figure 8), and the heat stress response element HSE was found in the promoter region of each of these genes (Table S2).

Furthermore, the CBL-CIPK complex also has been shown to regulate plant growth in response to abiotic stresses. The CBL1-CIPK6 component plays an important role in the plant response to high salinity, phosphorous deficiency and ABA signaling in *Brassica napus* (Chen et al., 2012). Both PtCBL10A and PtCBL10B could regulate poplar salt tolerance via interacting with PtSOS2 (Tang et al., 2013). AtCBL2 and AtCBL3 could recruit AtCIPK21 to the tonoplast and regulate *Arabidopsis* response to osmotic or salt stress (Pandey et al., 2015). The activity of AKT1 was also regulated by CIPK6 or CIPK16 in a CBL1-dependent manner (Lee et al., 2007). AtCIPK24 interaction with AtCBL4 or AtCBL10 regulates the activity of AtSOS1 to enhance *Arabidopsis* salt tolerance (Quan et al., 2007; Quintero et al., 2011). *MeCBL4* and *MeCBL10*, homologous with *AtCBL4* and *AtCBL10*, were induced by salt stress in roots and leaves, respectively (Figure 6), *MeCIPK24*, orthologous with *AtCIPK24*, was up-regulated in roots under salt treatment (Figure 7). The yeast two-hybrid test showed that MeCIPK24 could interact with MeCBL10 (Table 2, Figure S4). Yeast cells co-expressing *MeCBL10, MeCIPK24*, and *MeSOS1* showed enhanced salt tolerance compared with cells that have just expression of *MeSOS1* or co-expression of *MeCIPK24* and *MeSOS1* (Figure 9), which suggests that MeCIPK24 interaction with MeCBL10 could regulate the activity of MeSOS1 in yeast cells. MeCIPK24 interacted with MeCBL2 and MeCBL6 in addition to MeCBL10 (Table 2, Figure S4). *MeCBL6* was mainly induced by salt stress in roots (Figure 6), and demonstrated similar expression patterns with *MeCIPK24* (Figure 7), which suggests that the MeCIPK24-MeCBL6 complex might play a role in regulating salt tolerance in cassava. Upon cold stress, the expression level of MeCIPK7 showed the biggest change (Figure 7), and MeCIPK7 interacted with MeCBL2, MeCBL4 and MeCBL6 (Table 2, Figure S4). *MeCBL2* was mainly induced by cold stress and showed a similar expression profile to *MeCIPK7*, which suggests that the MeCIPK7-MeCBL2 complex might be involved in cold signal transduction. Under the treatment of heat, the expression level of *MeCIPK17* had the biggest change, the expression level reached a peak of 91-fold after 9 h of treatment (Figure 8), and MeCIPK17 interacted with MeCBL5 (Table 2, Figure S4), which suggests that the MeCIPK17-MeCBL5 complex might be involved in the plant response to heat stress. As shown in Figures 6, 7, *MeCBL4, MeCBL10*, and *MeCIPK19* were induced by PEG, which suggests that MeCIPK19 might regulate drought tolerance through interaction with MeCBL4 or MeCBL10.

**Figure 10:**
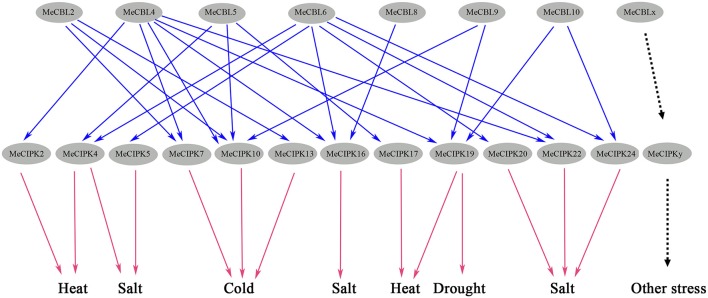
Potential CBL-CIPK signaling networks in cassava response to abiotic stresses. Blue arrows represent the interaction relationships between MeCBL and MeCIPK proteins, as indicated by protein-protein interaction data and red arrows represent observed gene expression responses to abiotic stresses.

In summary, the expression of *MeCBLs* and *MeCIPKs* in response to salt stress, drought, high and low temperature stress and tissue development was very diverse, but induction was observed in each stress treatment. Different MeCBLs could interact with one or more MeCIPKs (Table 2, Figure S4), indicating that various MeCBLs and MeCIPKs may participate in the signal transduction response to these stresses (Figure 10).

## Author contributions

XJ and YZ conceived and designed the experiments; CM, SW, YX, and NR performed the experiments; YZ, CM, and SW analyzed the data; XJ contributed reagents, materials, analysis tools; YZ and XJ wrote the paper.

### Conflict of interest statement

The authors declare that the research was conducted in the absence of any commercial or financial relationships that could be construed as a potential conflict of interest.

## Supplementary material

Figure S1 Phylogenetic relationships of cassava CBL proteins were compared with *Arabidopsis* and rice CBL family proteins. The Neighbor-Joining phylogenetic tree was constructed using MEGA 5.0 software with bootstrap value 1000 based on the amino acid sequences of CBL proteins from representative species. The cassava CBL proteins (MeCBL) are marked by triangle. The *Arabidopsis* CBL proteins (AtCBL) are marked by dots. The rice CBL proteins (OsCBL) are marked by square.

Figure S2 Phylogenetic relationships of cassava CIPK proteins were compared with *Arabidopsis* and rice CIPK family proteins. The Neighbor-Joining phylogenetic tree was constructed using MEGA 5.0 software with bootstrap value 1,000 based on the amino acid sequences of CIPK proteins from representative species. The cassava CIPK proteins (MeCIPK) are marked by triangles. The *Arabidopsis* CIPK proteins (AtCIPK) are marked by dots. The rice CIPK proteins (OsCIPK) are marked by square.

Figure S3 The amino acid sequences of each motif identified in CBL **(A)** and CIPK **(B)** proteins. The conserved motifs were identified using the MEME program. Width represents the number of each motif.

Figure S4 The interaction analysis of cassava MeCBL and MeCIPK proteins were performed using the Y2H method. The *MeCBL* genes were inserted into the pGBKT7 vector and the *MeCIPK* genes were cloned into the the pGADT7 vector. Yeast cells co-transformed with *MeCBL* and *MeCIPK* were grown on non-selective (lacking Leu and Trp, DDO) or selective media containing 40 μg/mL X-α-gal and 125 ng/mL Aureobasidin A (lacking Leu, Trp, His and Ade, QDO/X/A).

Table S1 Primers used in this study.

Table S2 Promoter analysis of *MeCBL* and *MeCIPK* genes. Types of potential *cis*-acting elements along with their functions (left) were identified in the 2,000 bp up-stream regions of 8 CBL genes and 26 CIPK genes (top).

## References

[B1] Albrecht V. Ritz O. Linder S. Harter K. Kudla J. (2001). The NAF domain defines a novel protein-protein interaction module conserved in Ca^2+^-regulated kinases. EMBO J. 20, 1051–1063. 10.1093/emboj/20.5.1051 11230129 PMC145464

[B2] Albrecht V. Weinl S. Blazevic D. D'Angelo C. Batistic O. Kolukisaoglu U.. (2003). The calcium sensor CBL1 integrates plant responses to abiotic stresses. Plant J. 36, 457–470. 10.1046/j.1365-313X.2003.01892.x 14617077

[B3] Batistic O. Kudla J. (2009). Plant calcineurin B-like proteins and their interacting protein kinases. Biochim. Biophy. Acta 1793, 985–992. 10.1016/j.bbamcr.2008.10.006 19022300

[B4] Chen L. Ren F. Zhou L. Wang Q. Q. Zhong H. Li X. B. (2012). The *Brassica napus* calcineurin B-like 1/CBL-interacting protein kinase 6 (CBL1/CIPK6) component is involved in the plant response to abiotic stress and ABA signaling. J. Exp. Bot. 63, 6211–6222. 10.1093/jxb/ers273 23105131 PMC3481211

[B5] Chen X. F. Gu Z. M. Xin D. D. Hao L. A. Liu C. J. Huang J.. (2011). Identification and characterization of putative *CIPK* genes in maize. J. Genet. Genomics 38, 77–87. 10.1016/j.jcg.2011.01.005 21356527

[B6] Cheng N. H. Pittman J. K. Zhu J. K. Hirschi K. D. (2004). The protein kinase SOS2 activates the *Arabidopsis* H^+^/Ca2^+^ antiporter CAX1 to integrate calcium transport and salt tolerance. J. Biol. Chem. 279, 2922–2926. 10.1074/jbc.M309084200 14583601

[B7] Cheong Y. H. Sung S. J. Kim B. G. Pandey G. K. Cho J. S. Kim K. N. (2010). Constitutive overexpression of the calcium sensor CBL5 confers osmotic or drought stress tolerance in *Arabidopsis*. Mol. Cells 29, 159–165. 10.1007/s10059-010-0025-z 20077023

[B8] Drerup M. M. Schlucking K. Hashimoto K. Manishankar P. Steinhorst L. Kuchitsu K.. (2013). The calcineurin B-like calcium sensors CBL1 and CBL9 together with their interacting protein kinase CIPK26 regulate the *Arabidopsis* NADPH oxidase RBOHF. Mol. Plant 6, 559–569. 10.1093/mp/sst009 23335733

[B9] Du W. M. Lin H. X. Chen S. Wu Y. S. Zhang J. Fuglsang A. T.. (2011). Phosphorylation of SOS3-like calcium-binding proteins by their interacting SOS2-like protein kinases is a common regulatory mechanism in *Arabidopsis*. Plant Physiol. 156, 2235–2243. 10.1104/pp.111.173377 21685179 PMC3149935

[B10] Fuglsang A. T. Guo Y. Cuin T. A. Qiu Q. Song C. Kristiansen K. A.. (2007). *Arabidopsis* protein kinase PKS5 inhibits the plasma membrane H^+^ -ATPase by preventing interaction with protein. Plant Cell 19, 1617–1634. 10.1105/tpc.105.035626 17483306 PMC1913743

[B11] Guo W. Chen T. Hussain N. Zhang G. Jiang L. (2016). Characterization of salinity tolerance of transgenic rice lines harboring *HsCBL8* of wild barley (*Hordeum spontanum*) line from Qinghai-Tibet plateau. Front. Plant Sci. 7:1678. 10.3389/fpls.2016.01678 27891136 PMC5102885

[B12] Guo Y. Halfter U. Ishitani M. Zhu J. K. (2001). Molecular characterization of functional domains in the protein kinase SOS2 that is required for plant salt tolerance. Plant Cell 13, 1383–1400. 10.1105/tpc.13.6.1383 11402167 PMC135579

[B13] Ho C. Lin S. Hu H. Tsay Y. (2009). CHL1 functions as a nitrate sensor in plants. Cell 138, 1184–1194. 10.1016/j.cell.2009.07.004 19766570

[B14] Hrabak E. M. Chan C. W. M. Gribskov M. Harper J. M. Choi J. H. Halford N. G.. (1996). Characterization of eight new members of the calmodulin-like domain protein kinase gene family from *Arabidopsis thaliana*. Plant Mol. Biol. 31, 405–412. 10.1007/BF00021802 8756605

[B15] Hu H. C. Wang Y. Y. Tsay Y. F. (2009). AtCIPK8, a CBL-interacting protein kinase, regulates the low-affinity phase of the primary nitrate response. Plant J. 57, 264–278. 10.1111/j.1365-313X.2008.03685.x 18798873

[B16] Huang C. Ding S. Zhang H. Du H. An L. (2011). CIPK7 is involved in cold response by interacting with CBL1 in *Arabidopsis thaliana*. Plant Sci. 181, 57–64. 10.1016/j.plantsci.2011.03.011 21600398

[B17] Kim K. N. Cheong Y. H. Grant J. J. Pandey G. K. Luan S. (2003). CIPK3, a calcium ensorassociated protein kinase that regulates abscisic acid and cold signal transduction in *Arabidopsis*. Plant Cell 15, 411–423. 10.1105/tpc.006858 12566581 PMC141210

[B18] Kolukisaoglu U. Weinl S. Blazevic D. Batistic O. Kudal J. (2004). Calcium sensors and their interacting protein kinases: genomics of the *Arabidopsis* and rice CBL-CIPK signaling networks. Plant Physiol. 134, 43–58. 10.1104/pp.103.033068 14730064 PMC316286

[B19] Kudla J. Batistic O. Hashimoto K. (2010). Calcium signals: the lead currency of plant information processing. Plant Cell 22, 541–563. 10.1105/tpc.109.072686 20354197 PMC2861448

[B20] Lee S. C. Lan W. Z. Kim B. G. Li L. Cheong Y. H. Pandey G. K.. (2007). A protein phosphorylation/dephosphorylation network regulates a plant potassium channel. Proc. Natl. Acad. Sci. U.S.A. 104, 15959–15964. 10.1073/pnas.0707912104 17898163 PMC2000415

[B21] Liu J. Y. Chen N. N. Chen F. Cai B. Dal Santo S. Tornielli G. B.. (2014). Genome-wide analysis and expression profile of the bZIP transcription factor gene family in grapevine (*Vitis vinifera*). BMC Genomics 15:281. 10.1186/1471-2164-15-281 24725365 PMC4023599

[B22] Liu L. L. Ren H. M. Chen L. Q. Wang Y. Wu W. H. (2013). A protein kinase, calcineurin B-like protein-interacting protein kinase 9, interacts with calcium sensor calcineurin B-like protein 3 and regulates potassium homeostasis under low-potassium stress in *Arabidopsis*. Plant Physiol. 161, 266–277. 10.1104/pp.112.206896 23109687 PMC3532257

[B23] Luan S. (2009). The CBL-CIPK network in plant calcium signaling. Trenes Plant Sci. 14, 37–42. 10.1016/j.tplants.2008.10.005 19054707

[B24] Luan S. Kudla J. Rodriguez-Concepcion M. Yalovsky S. Gruissem W. (2002). Calmodulins and calcineurin B-like proteins: calcium sensors for specific signal response coupling in plants. Plant Cell 14, S389–S400. 10.1105/tpc.001115 12045290 PMC151268

[B25] Luan S. Lan W. Z. Lee S. C. (2009). Potassium nutrition, sodium toxicity, and calcium signaling: connections through the CBL-CIPK network. Curr. Opin. Plant Biol. 12, 339–346. 10.1016/j.pbi.2009.05.003 19501014

[B26] Ma B. J. Gu Z. M. Tang H. J. Chen X. F. Liu F. Zhang H. S. (2010). Preliminary study on function of calcineurin B-like protein gene *OsCBL8* in Rice. Rice Sci. 17, 10–18. 10.1016/S1672-6308(08)60099-2

[B27] Martinez-Atienza J. Jiang X. Y. Garciadeblas B. Mendoza I. Zhu J. K. Pardo J. M.. (2007). Conservation of the salt overly sensitive pathway in rice. Plant Physiol. 143, 1001–1012. 10.1104/pp.106.092635 17142477 PMC1803719

[B28] Nagae M. Nozawa A. Koizumi N. Sano H. Hashimoto H. Sato M.. (2003). The crystal structure of the novel calcium-binding protein AtCBL2 from *Arabidopsis thaliana*. J. Biol. Chem. 278, 42240–42246. 10.1074/jbc.M303630200 12871972

[B29] Ohta M. Guo Y. Halfter U. Zhu J. K. (2003). A novel domain in the protein kinase SOS2 mediates interaction with the protein phosphatase 2C ABI2. Proc. Natl. Acad. Sci. U.S.A. 100, 11771–11776. 10.1073/pnas.2034853100 14504388 PMC208833

[B30] Oliveira E. J. Santana F. A. Oliveira L. A. Santos V. S. (2014). Genetic parameters and prediction of genotypic values for root quality traits in cassava using REML/BLUP. Genet. Mol. Res. 13, 6683–6700. 10.4238/2014.August.28.13 25177949

[B31] Pandey G. K. Grant J. J. Cheong Y. H. Kim B. G. Li L. G. Luan S. (2008). Calcineurin-B-like protein CBL9 interacts with target kinase CIPK3 in the regulation of ABA response in seed germination. Mol. Plant 1, 238–248. 10.1093/mp/ssn003 19825536

[B32] Pandey G. K. Kanwar P. Singh A. Steinhorst L. Pandey A. Yadav A. K. (2015). CBL-interacting protein kinase, CIPK21, regulates osmotic and salt stress responses in *Arabidopsis*. Plant Physiol. 169, 780–792. 10.1104/pp.15.00623 26198257 PMC4577403

[B33] Qiu Q. S. Guo Y. Quintero F. J. Pardo J. M. Schumaker K. S. Zhu J. K. (2004). Regulation of vacuolar Na^+^/H^+^ exchange in *Arabidopsis thaliana* by the salt-overly-sensitive (SOS) pathway. J. Biol. Chem. 279, 207–215. 10.1074/jbc.M307982200 14570921

[B34] Quan R. Lin H. X. Mendoza I. Zhang Y. G. Cao W. H. Yang Y. Q.. (2007). SCABP8/CBL10, a putative calcium sensor, interacts with the protein kinase SOS2 to protect *Arabidopsis* shoots from salt stress. Plant Cell 19, 1415–1431. 10.1105/tpc.106.042291 17449811 PMC1913747

[B35] Quintero F. J. Martinez-Atienza J. Villalta I. Jiang X. Y. Kim W. Y. Ali Z.. (2011). Activation of the plasma membrane Na/H antiporter SOS1 by phosphorylation of an auto-inhibitory C-terminal domain. Proc. Natl. Acad. Sci. U.S.A. 108, 2611–2616. 10.1073/pnas.1018921108 21262798 PMC3038701

[B36] Ren X. L. Qi G. N. Feng H. Q. Zhao S. Zhao S. S. Wang Y.. (2013). Calcineurin B-like protein CBL10 directly interacts with AKT1 and modulates K^+^ homeostasis in *Arabidopsis*. Plant J. 74, 258–266. 10.1111/tpj.12123 23331977

[B37] Sanchez-Barrena M. J. Martinez-Ripoll M. Zhu J. K. Albert A. (2005). The structure of the *Arabidopsis thaliana* SOS3: molecular mechanism of sensing calcium for salt stress response. J. Mol. Biol. 345, 1253–1264. 10.1016/j.jmb.2004.11.025 15644219

[B38] Sanders D. Pelloux J. Brownlee C. Harper J. F. (2002). Calcium at the crossroads of signaling. Plant Cell 14, S401–S417. 10.1105/tpc.002899 12045291 PMC151269

[B39] Shukla V. Mattoo A. K. (2008). Sucrose non-fermenting 1-related protein kinase 2 (SnRK2): a family of protein kinases involved in hyperosmotic stress signaling. Physiol. Mol. Biol. Plants 14, 91–100. 10.1007/s12298-008-0008-0 23572876 PMC3550663

[B40] Sun T. Wang Y. Wang M. Li T. T. Zhou Y. Wang X. T.. (2015). Identification and comprehensive analyses of the *CBL* and *CIPK* gene families in wheat (*Triticum aestivem* L.). BMC Plant Biol. 15:269. 10.1186/s12870-015-0657-4 26537110 PMC4634908

[B41] Tang R. J. Liu H. Bao Y. Lv Q. D. Yang L. Zhang H. X. (2010). The woody plant poplar has a functionally conserved salt overly sensitive pathway in response to salinity stress. Plant Mol. Biol. 74, 367–380. 10.1007/s11103-010-9680-x 20803312

[B42] Tang R. J. Yang Y. Yang L. Liu H. Wang C. T. Yu M. M.. (2013). Poplar calcineurin B-like proteins PtCBL10A and PtCBL10B regulate shoot salt tolerance through interaction with PtSOS2 in the vacuolar membrane. Plant Cell Environ. 37, 573–588. 10.1111/pce.12178 23941462

[B43] Tang R. J. Zhao F. G. Garcia V. J. Kleist T. J. Yang L. Zhang H. X.. (2015). Tonoplast CBL-CIPK calcium signaling network regulates magnesium homeostasis in *Arabidopsis*. Proc. Natl. Acad. Sci. U.S.A. 112, 3134–3139. 10.1073/pnas.1420944112 25646412 PMC4364200

[B44] Wang N. Zheng Y. Xin H. P. Fang L. C. Li S. H. (2013). Comprehensive analysis of NAC domain transcription factor gene family in *Vitis vinifera*. Plant Cell Rep. 32, 61–75. 10.1007/s00299-012-1340-y 22983198

[B45] Wang X. P. Chen L. M. Liu W. X. Shen L. K. Wang F. L. Zhou Y. (2016). AtKC1 and CIPK12 synergistically modulate AKT1-mediated low-potassium stress responses in *Arabidopsis*. Plant Physiol. 170, 2264–2277. 10.1104/pp.15.01493 26829980 PMC4825127

[B46] Xi Y. Liu J. Dong C. Cheng Z. M. (2017). The, C. B. L., and CIPK gene family in grapevine (Vitis vinifera): genome-wide analysis and expression profiles in response to various abiotic stresses. Front. Plant Sci. 8:978 10.3389/fpls.2017.00978 28649259 PMC5465270

[B47] Xiang Y. Huang Y. Xiong L. A. (2007). Characterization of stress responsive *CIPK* genes in rice for stress tolerance improvement. Plant Physiol. 144, 1416–1428. 10.1104/pp.107.101295 17535819 PMC1914128

[B48] Xu J. Li H. D. Chen L. Q. Wang Y. Liu L. L. He L.. (2006). A protein kinase, interacting with two calcineurin B-like proteins, regulates K^+^ transporter AKT1 in *Arabidopsis*. Cell 125, 1347–1360. 10.1016/j.cell.2006.06.011 16814720

[B49] Yu Q. An L. Li W. (2014). The CBL-CIPK network mediates different signaling pathways in plants. Plant Cell Rep. 33, 203–214. 10.1007/s00299-013-1507-1 24097244

[B50] Yu Y. H. Xia X. L. Yin W. L. Zhang H. X. (2007). Comparative genomic analysis of *CIPK* gene family in *Arabidopsis* and *Populus*. Plant Growth Regul. 52, 101–110. 10.1007/s10725-007-9165-3

[B51] Zeng C. Chen Z. Xia J. Zhang K. Chen X. Zhou Y.. (2014). Chilling acclimation provides immunity to stress by altering regulatory networks and inducing genes with protective functions in cassava. BMC Plant Biol. 14:207. 10.1186/s12870-014-0207-5 25090992 PMC4236759

[B52] Zhai J. L. Xu H. X. Cong X. L. Deng Y. C. Xia Z. H. Huang X. (2013). Ca^2+^/H^+^ exchange in the plasma membrane of *Arabidospsis thaliana* leaves. Acta Physiol. Plant 35, 161–173. 10.1007/s11738-012-1059-y

[B53] Zhang H. C. Yin W. L. Xia X. L. (2008). Calcineurin B-like family in Populus: comparative genome analysis and expression pattern under cold, drought and salt stress treatment. Plant Growth Regul. 56, 129–140. 10.1007/s10725-008-9293-4

[B54] Zhang H. F. Yang B. Liu W. Z. Li H. W. Wang L. Wang B. Y.. (2014). Identification and characterization of *CBL* and *CIPK* gene families in canola (*Brassica napus* L.). BMC Plant Biol. 14:8. 10.1186/1471-2229-14-8 24397480 PMC3890537

[B55] Zhou Y. Yin X. C. Duan R. J. Hao G. P. Guo J. C. Jiang X. Y. (2015). SpAHA1 and SpSOS1 coordinate in transgenic yeast to improve salt tolerance. PLoS ONE 10:e0137447. 10.1371/journal.pone.0137447 26340746 PMC4560418

[B56] Zhu J. K. (2003). Regulation of ion homeostasis under salt stress. Curr. Opin. Plant Biol. 6, 441–445. 10.1016/S1369-5266(03)00085-2 12972044

[B57] Zhu K. Chen F. Liu J. Chen X. Hewezi T. Cheng Z. M. (2016). Evolution of an intron-poor cluster of the *CIPK* gene family and expression in response to drought stress in soybean. Sci. Rep. 6:28225. 10.1038/srep28225 27311690 PMC4911590

