# Supplementary figures and images for: Expression Patterns and Identified Protein-Protein Interactions Suggest That Cassava CBL-CIPK Signal Networks Function in Responses to Abiotic Stresses
Source: Front Plant Sci. 2018 Mar 2;9:269. doi: 10.3389/fpls.2018.00269 (PMC5841119; doi:10.3389/fpls.2018.00269)

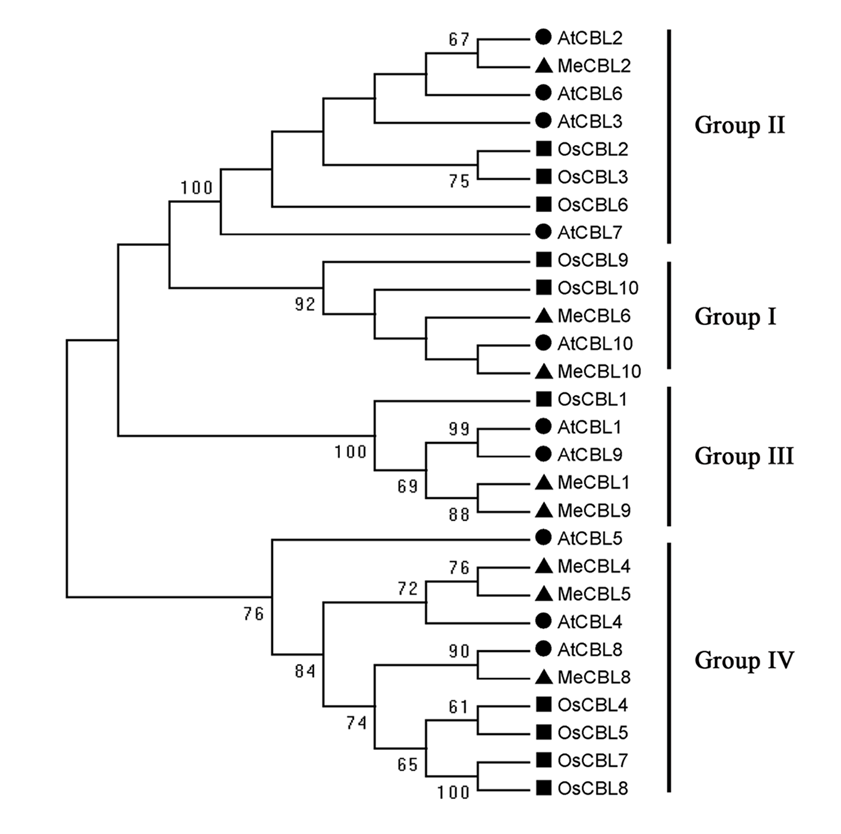

Supplement: Figure S1 — Phylogenetic relationships of cassava CBL proteins were compared with Arabidopsis and rice CBL family proteins. The Neighbor-Joining phylogenetic tree was constructed using MEGA 5.0 software with bootstrap value 1000 based on the amino acid sequences of CBL proteins from representative species. The cassava CBL proteins (MeCBL) are marked by triangle. The Arabidopsis CBL proteins (AtCBL) are marked by dots. The rice CBL proteins (OsCBL) are marked by square. [file Image1.TIF]

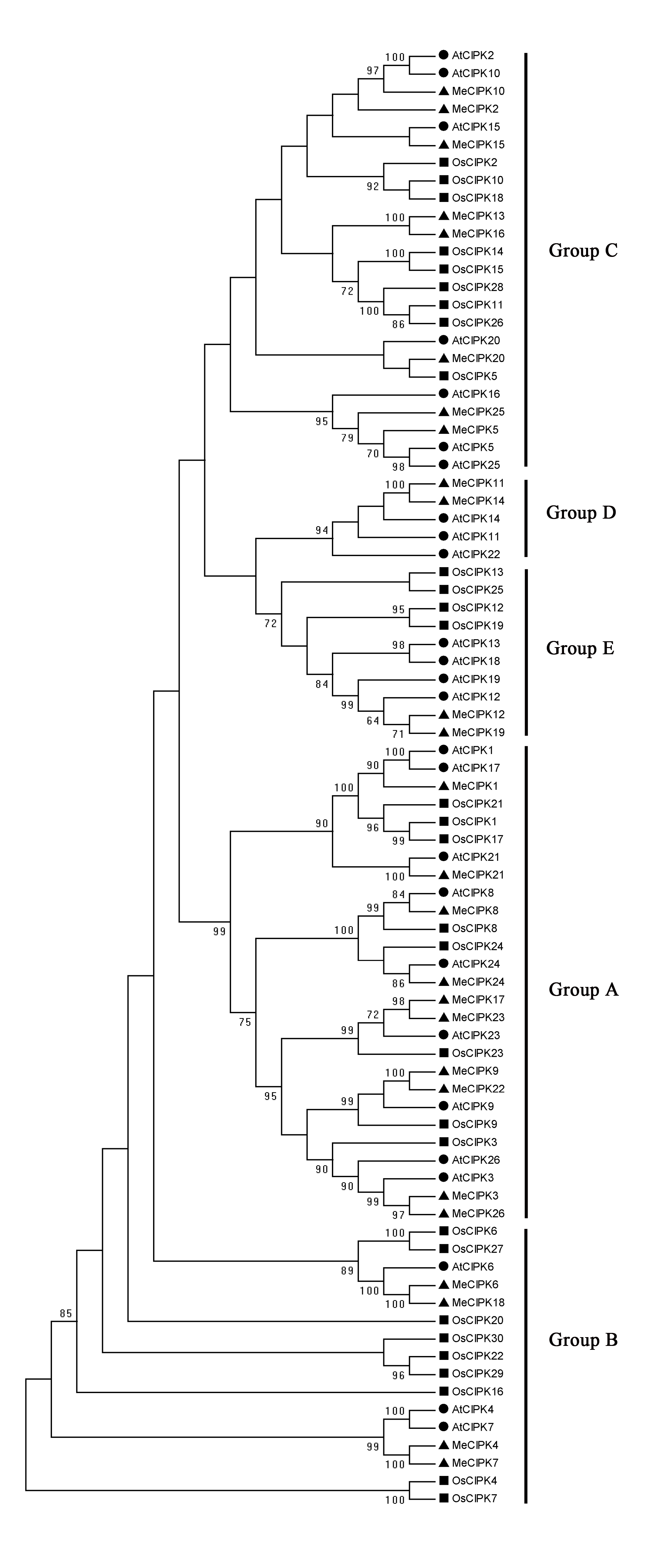

Supplement: Figure S2 — Phylogenetic relationships of cassava CIPK proteins were compared with Arabidopsis and rice CIPK family proteins. The Neighbor-Joining phylogenetic tree was constructed using MEGA 5.0 software with bootstrap value 1,000 based on the amino acid sequences of CIPK proteins from representative species. The cassava CIPK proteins (MeCIPK) are marked by triangles. The Arabidopsis CIPK proteins (AtCIPK) are marked by dots. The rice CIPK proteins (OsCIPK) are marked by square. [file Image2.TIF]

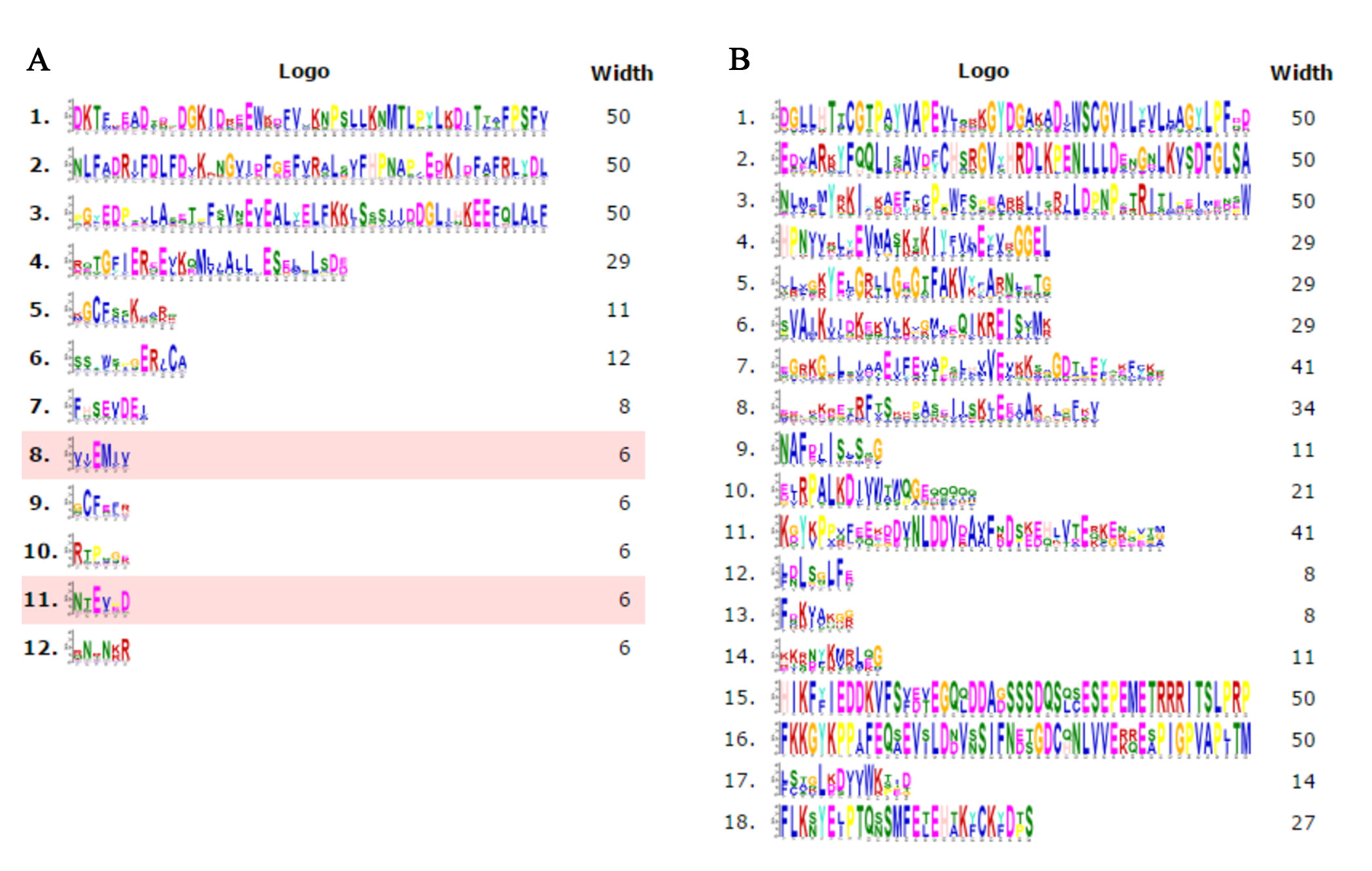

Supplement: Figure S3 — The amino acid sequences of each motif identified in CBL (A) and CIPK (B) proteins. The conserved motifs were identified using the MEME program. Width represents the number of each motif. [file Image3.TIF]

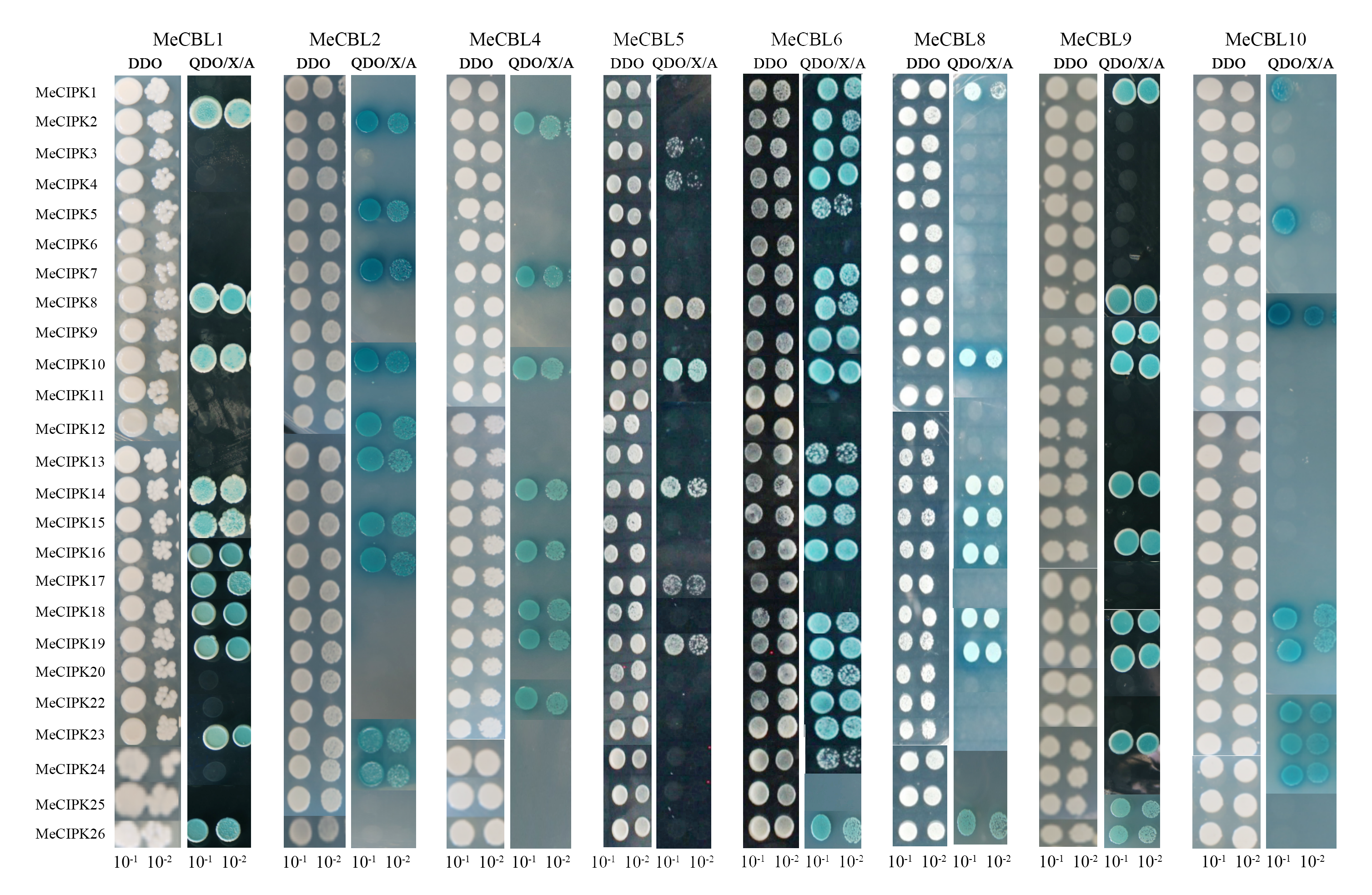

Supplement: Figure S4 — The interaction analysis of cassava MeCBL and MeCIPK proteins were performed using the Y2H method. The MeCBL genes were inserted into the pGBKT7 vector and the MeCIPK genes were cloned into the the pGADT7 vector. Yeast cells co-transformed with MeCBL and MeCIPK were grown on non-selective (lacking Leu and Trp, DDO) or selective media containing 40 μg/mL X-α-gal and 125 ng/mL Aureobasidin A (lacking Leu, Trp, His and Ade, QDO/X/A). [file Image4.TIF]
